# Unveiling the emerging role of curcumin to alleviate ochratoxin A-induced muscle toxicity in grass carp (*Ctenopharyngodon idella*): in vitro and in vivo studies

**DOI:** 10.1186/s40104-024-01023-6

**Published:** 2024-05-12

**Authors:** Piao Zhao, Lin Feng, Weidan Jiang, Pei Wu, Yang Liu, Hongmei Ren, Xiaowan Jin, Lu Zhang, Haifeng Mi, Xiaoqiu Zhou

**Affiliations:** 1 Animal Nutrition Institute, Sichuan Agricultural University, Chengdu, 611130 Sichuan China; 2 Fish Nutrition and Safety Production University Key Laboratory of Sichuan Province, Sichuan Agricultural University, Chengdu, 611130 Sichuan China; 3 Key Laboratory of Animal Disease-Resistance Nutrition, Ministry of Education, Ministry of Agriculture and Rural Affairs, Key Laboratory of Sichuan Province, Chengdu, 611130 Sichuan China; 4 Tongwei Co., Ltd., Healthy Aquaculture Key Laboratory of Sichuan Province, Chengdu, 610041 Sichuan China

**Keywords:** Curcumin, Grass carp, Myotoxic, Ochratoxin A, Protein deposition

## Abstract

**Background:**

Ochratoxin A (OTA), a globally abundant and extremely hazardous pollutant, is a significant source of contamination in aquafeeds and is responsible for severe food pollution. The developmental toxicity of OTA and the potential relieving strategy of natural products remain unclear. This study screened the substance curcumin (Cur), which had the best effect in alleviating OTA inhibition of myoblast proliferation, from 96 natural products and investigated its effect and mechanism in reducing OTA myotoxicity in vivo and in vitro.

**Methods:**

A total of 720 healthy juvenile grass carp, with an initial average body weight of 11.06 ± 0.05 g, were randomly assigned into 4 groups: the control group (without OTA and Cur), 1.2 mg/kg OTA group, 400 mg/kg Cur group, and 1.2 mg/kg OTA + 400 mg/kg Cur group. Each treatment consisted of 3 replicates (180 fish) for 60 d.

**Results:**

Firstly, we cultured, purified, and identified myoblasts using the tissue block culture method. Through preliminary screening and re-screening of 96 substances, we examined cell proliferation-related indicators such as cell viability and ultimately found that Cur had the best effect. Secondly, Cur could alleviate OTA-inhibited myoblast differentiation and myofibrillar development-related proteins (MyoG and MYHC) in vivo and in vitro and improve the growth performance of grass carp. Then, Cur could also promote the expression of OTA-inhibited protein synthesis-related proteins (S6K1 and TOR), which was related to the activation of the AKT/TOR signaling pathway. Finally, Cur could downregulate the expression of OTA-enhanced protein degradation-related genes (*murf1*, *foxo3a*, and *ub*), which was related to the inhibition of the FoxO3a signaling pathway.

**Conclusions:**

In summary, our data demonstrated the effectiveness of Cur in alleviating OTA myotoxicity in vivo and in vitro. This study confirms the rapidity, feasibility, and effectiveness of establishing a natural product screening method targeting myoblasts to alleviate fungal toxin toxicity.

**Graphical Abstract:**

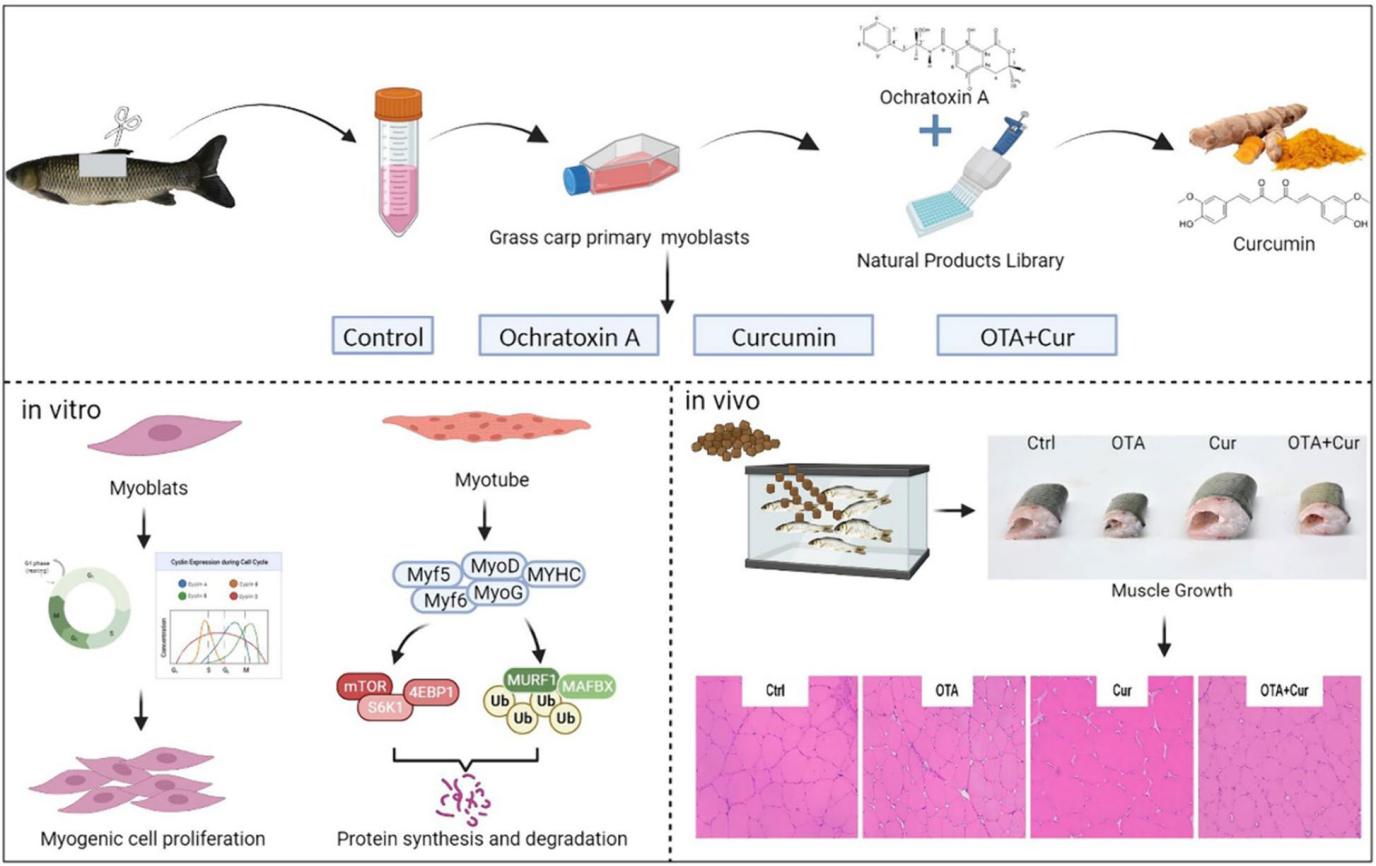

**Supplementary Information:**

The online version contains supplementary material available at 10.1186/s40104-024-01023-6.

## Introduction

As one of the most hazardous and prevalent mycotoxins in the world, ochratoxin A (OTA) is a mycotoxin produced by fungi as a secondary metabolite [1], and according to the International Agency for Research on Cancer (IARC), OTA belongs to the group 2B carcinogens [2]. OTA mainly contaminates foods such as coffee, wine and meat products, as well as feed and feed ingredients [3, 4]. A previous study showed that OTA occurred in 95% of fish feed samples [5]. Evidence has shown that OTA-contaminated diets decrease fish growth [6, 7], induce liver [7], spleen [8], and muscle damage [9, 10]. However, being the beneficial role of food welfare for human beings, e.g., muscles, whether OTA-polluted diets have impacts on muscle lesions or abnormal development of muscle fibers is unknown. Besides, ecotechnology methods to address the issues of myotoxicity alleviation have already become mainstream [11], especially with the addition of natural products [12, 13], because most of those mycotoxins could not be wholly eliminated by industrial processes (including heat treatment and microbial degradation) [11, 14]. However, systematic screening of natural products effectively to mitigate or prevent OTA-induced animal growth and muscle developmental toxicity, as well as underlying mechanisms integrated into in vivo and in vitro studies, still lacks attention.

Generally, animal muscle growth typically involves a combination of hyperplasia, which is an increase in muscle fiber number, and hypertrophy, which is an increase in muscle fiber size. In mammals, hypertrophic growth occurs after birth, while in fish muscle growth differs in this regard [15]. On the one hand, cell proliferation, and differentiation are intricately linked to the regulation of the cell cycle proteins such as cyclin B, cyclin D and cyclin E, E2F transcription factor 4 (E2F4), proliferating cell nuclear antigen (PCNA) [16, 17] and myoblast determination protein (MyoD), myogenin (MyoG), and myosin heavy chain (MYHC) [18–20]. To date, there is a lack of research on the impact of OTA on the expression of these cell cycle proteins, MyoD, MyoG, and MYHC in animal muscle. Qi et al. [21] reported a decrease in rat liver PCNA, HK-2 cell cyclin D and E [22], and chicken kidney MYHC [23] expression in response to OTA. A study on MDCK-C7 cells has shown that OTA treatment could inhibit the response to insulin-like growth factor 1 (IGF1) [24]. In the IGF-1 knockout mouse muscle, the mRNA expression of *myog *was significantly lower [25]. These studies implied that OTA might inhibit the cell cycle and MyoD and MyoG in animal muscle development. On the other hand, myoblast fusion is associated with protein deposition [26]. The targets of rapamycin/forkhead box O (TOR/FoxO) regulate protein synthesis and degradation, respectively [27]. Previous studies in hepatoma cells [28], HEK 293 cells [29, 30], and rat liver [31] have shown OTA inhibits protein synthesis. In PK15 cells, OTA could inhibit the phosphorylation of TOR [32]. Protein degradation primarily relies on the ubiquitin–proteasome system (UPS) [33]. Previous research in HK-2 and MEF cells showed that OTA exposure promoted ubiquitin (Ub) protein expression [34]. Protein kinase B (AKT) phosphorylates FoxO transcription factors (TF) and impedes their nuclear activity, thus suppressing FoxO-dependent atrogene expression [35]. It is certain that OTA could inhibit the phosphorylation of AKT in PK15 cells [32]. OTA may regulate protein synthesis and degradation via the TOR and FoxO signaling pathways, respectively. However, this needs to be further investigated.

Curcumin (Cur) is a natural product with antioxidant, anti-inflammatory, and anti-tumor properties [36]. It is also a pigment food additive and has received approval from both the Food and Agriculture Organization of the United Nations and China for its utilization in the food industry [37]. It was also included in China's "Catalogue of Feed Additives" in 2014 [38]. Previous studies have shown that adding Cur to the diet alleviates OTA induced intestinal immunotoxicity [39] and hepatotoxicity [40] in ducks, as well as nephrotoxicity in mice [41]. Although there is currently no research on Cur alleviating OTA induced fish toxicity, it has been found in mouse cardiac fibroblasts that adding Cur can reduce the proportion of G0/G1 phase and increase the expression of cyclin B, thereby inhibiting myocardial fibrosis [42]. In mouse muscle C2C12 cells, adding Cur significantly promotes myoblast differentiation and fusion, and increases the expression of MyoD and MyoG [43]. Injecting Cur into muscle atrophy mice significantly increases the expression of MyoD and MyoG, thereby improving muscle atrophy [44]. The above indicates that Cur may alleviate OTA induced muscle toxicity in grass carp by promoting muscle fiber development.

The growth of muscle in teleost fish is not predetermined at birth but rather follows a lifelong pattern characterized by myofiber hyperplasia and myofiber hypertrophy [45], which are dependent on the processes of myoblast proliferation, differentiation, and fusion [46]. It has been reported that OTA could contaminate over 80% of aquafeed [47], which not only brings huge economic losses to the aquaculture industry but also jeopardizes human health through food chain accumulation [48]. In this study, grass carp (*Ctenopharyngodon idella*), a significant economic species of fish, was utilized as a model. The hypothesis posited OTA may negatively affect the muscle growth of fish, potentially due to the development of muscle fibers. It was further suggested that these detrimental effects could be dismissed by Cur. To test this assumption, our primary objective was to explore the effect of Cur to mitigate the detrimental effects of feed pollutants or toxins on animal production. This investigation involved both in vivo and in vitro validation to address the pressing issue of mycotoxin contamination in animal feed. Additionally, this research contributes to the theoretical understanding of the mechanisms underlying the observed effects. It also provides a theoretical and practical basis for the development and application of natural products.

## Materials and methods

This study was approved by The Animal Care Advisory Committee of Sichuan Agricultural University (Chengdu, Sichuan, China) with license number ZP-2019114003.

### Primary myoblasts of grass carp (GCPMs) isolation and culture

According to He et al. [49], the tissue block culture method was performed. After anesthesia for ~ 50 g of healthy grass carp, sterilizing the body surface with alcohol cotton balls, gently scraping off scales on the side of the grass carp, and taking 2 g of muscle tissue for dissociation into small pieces of approximately 1 mm^3^. The tissue fragments were then transferred into a centrifuge tube, followed by the addition of 20 mL of DMEM-F12 medium (Gibco, Carlsbad, CA, USA) using a pipette. The muscle tissue fragments were purged with the pipette and repeated 10 times. The tissue fragments were transferred into a 25-mL cell culture flask with 1 mL DMEM-F12 medium, and then cultured in a closed manner at 28 °C in an incubator for 4 h until all the cells were attached to the flask. After that, discard the old cell culture medium and refresh with the addition of 5 mL of M199 complete culture medium [10% fetal bovine serum (FBS); 10 ng/mL basic fibroblast growth factor (bFGF); and 20 ng/mL basic epidermal growth factor (bEGF) were purchased from Invitrogen Corporation (Carlsbad, CA, USA)] to culture the cells continuously for 14 d. During the cell cultivation, fluid was changed every 3 d till the cell confluency reached 60%–80%. Based on the distinct adhesion characteristics of myoblasts and fibroblast, myoblasts were purified by the differential adhesion method. Notably, to ensure the purity of the myoblasts, a differential adherent was performed after each passage, and the purity of myoblasts was determined by observing the number of fibroblasts adherents for 30 min. After purification to the third generation, it was observed that no cells adhered to the wall within 30 min, indicating successful isolation of pure myoblasts. The third-generation myoblasts were subsequently utilized in subsequent experiments, with their identity confirmed through Desmin immunofluorescence staining. Furthermore, we have drawn the cell growth curve with the counting method. The details were as follows: 5 × 10^5^/mL was cultured to inoculate GCPMs and then counted every 24 h for consecutive 7 d.

### OTA and natural product library (NPL) treatment

The information on OTA and NPL was provided in supplementary Table S1. The 10 mmol/L OTA solution was prepared in dimethyl sulfoxide (DMSO) and diluted in a culture medium to achieve the final concentrations of 0 (0.1% DMSO), 0.2, 0.4, 0.6, 0.8, 1, and 2 μmol/L, respectively. The content of DMSO in each treatment was 0.1%. The collected GCPMs (2 × 10^5^) were inoculated on a 96-well plate and cultured for 48 h. Then, the plates were incubated with 0 (0.1% DMSO), 0.2, 0.4, 0.6, 0.8, 1, and 2 μmol/L OTA for 24, 48 and 72 h, and then a cell counting kit-8 (CCK8) assay was performed. An NPL containing 96 substances at a concentration of 10 mmol/L was diluted into 2.5, 5, and 10 μmol/L, respectively, and cultured with 0.8 μmol/L OTA for 48 h. Further screening experiments were conducted by selecting different concentrations of LA (0, 2, 4, 6, 8, 10, 20, and 40 μmol/L), Cur (0, 1.5, 3, 6, 12, and 24 μmol/L) and SaA (0, 5, 10, 20, 40, 80, 160, and 320 μmol/L) for cell viability (CV) tests, which were analyzed by CCK8.

### CCK8 assay

According to the instructions, CCK8 detection was performed by adding 10 μL of CCK8 reagent to each well for 2 h before analysis. The absorbance of treated cells was compared with that of untreated control cells to determine the CV; CV = [(experimental hole − blank hole)/(control hole − blank hole)] × 100%.

### Myoblast differentiation experiment

Purified myoblasts were inoculated on 12-well plates and cultured until the growth density was about 85%–90% [50]. Then, followed by adding a differentiation medium (M199 basic medium + 2% horse serum + 1% triple antibody). After 3 d of culture, we added different concentrations of OTA (0, 0.5, 1, 2, 4, and 6 μmol/L), Cur (0, 2, 4, 8, 16, and 32 μmol/L) and OTA + Cur (2 μmol/L OTA + 0, 2, 4, 8, 16, and 32 μmol/L Cur). The expression of MyoG and MyoD was detected by western blot to determine the concentration of OTA inhibiting and Cur promoting myoblast differentiation. The MK-2206 (AKT inhibitor, SF2712, Beyotime, Shanghai, China) was dissolved in DMSO (0.1%) and was used at a concentration of 0, 1, 2, 3, 4, 6, 8, and 10 μmol/L for 48 h to detect the protein expression of p-AKT, respectively. Wortmannin (FoxO3a activator, SL-2052, MedChemExpress, New Jersey, USA) was dissolved in DMSO (0.1%) at a concentration of 0, 2, 4, 6, 8, 10, 20, and 40 μmol/L for the 48 h cell culture to detect the protein expression of FoxO3a, respectively.

### Immunofluorescence staining

The GCPMs and primary myotube cells (GCPMTs) were fixed in 4% paraformaldehyde for 0.5 h, followed by three washes with PBS for 5 min each time. The cells were then permeabilized with 0.5% triton X-100 at room temperature for 0.5 h and washed three times with PBS for 5 min each. Subsequently, the samples were blocked with 5% goat serum for 0.5 h and finally incubated overnight at 4 °C with primary antibodies (Desmin diluted 1:100 and MYHC diluted 1:5). Following this, the cells were washed three times with PBS for 5 min each and incubated at room temperature for 1 h with a second antibody according to the type of secondary antibody (diluted at 1:1,000). Cells were finally stained with 4',6-diamidino-2-phenylindole (DAPI) for 10 min at room temperature and covered with glass cover slides. Antibodies information is listed in Table S3. For the paraffin section of muscle tissue immunofluorescence staining, the 5 μmol/L slices were repaired with antigen repair solution (0.2 g citric acid + 1.5 g trisodium citrate diluted with 500 mL pure water), treated with 3% hydrogen peroxide, and then blocked. The blocking and subsequent steps consisted of the immune crown staining of cells. Immunofluorescence images were obtained using a DMI4000B inverted fluorescence microscope (Leica). The information on primary and secondary antibodies and DAPI is given in Table S1.

### In vivo experiments and muscle sample collection of grass carp

The source information for OTA is detailed in Table S1. The components of the basal diet can be found in Table S4. Grass carp *(Ctenopharyngodon idella)* were purchased from Deyang Aquatic Seeds Company (Sichuan, China). Prior research has demonstrated that the growth performance of juvenile grass carp is hindered, and muscle damage occurs when fed 1,200 μg/kg OTA [6, 10]. Feeding 400 mg/kg Cur can improve the growth performance of juvenile grass carp [51], so we chose two doses for subsequent experiments. Those cages were randomly allocated into four groups: the control group (without OTA and Cur), 1.2 mg/kg OTA, 400 mg/kg Cur, and 1.2 mg/kg OTA plus 400 mg/kg Cur. After a 28-day acclimation period [52], a total of 720 3-month-old grass carp regardless of gender (initial weight: 11.06 ± 0.05 g) randomly distributed 12 aquaculture cages (1.5 m × 0.8 m × 1.5 m). Each treatment consisted of 3 replicates, with 60 fish in each replicate. The preparation method for feed containing OTA was based on previous research [10]. For the Cur feed supplement, first prepare a first-level premix at a ratio of 10 times larger (e.g., for 10 kg of feed, 4.2 g of Cur (95%) required 42.105 g of Cur + 457.895 g of microcrystalline cellulose), and then add 100 g of microcrystalline cellulose to the first level premix of Cur. The water temperature and pH value were measured at 27.8 ± 3 °C and 7.5 ± 0.4, respectively, with a dissolved oxygen level of ≥ 6.0 mg/L. Nitrite and ammonia concentrations were found to be in the ranges of 0.005–0.010 mg/L and 0.2–0.4 mg/L, respectively. The cages were situated in outdoor freshwater ponds and microporous aeration was used employed duration of the study. Water was changed on a daily basis in a consistent and measured manner, with feeding occurring five times per day (8:00, 11:00, 13:00, 15:00 and 19:00). Before the trial, they were fed a commercial diet at a rate of 4% of the initial body weight for 4 weeks to acclimatize to the experimental environment.

Following the conclusion of the growth trial, juveniles specimens underwent a 24-h fasting period before being with a 50 mg/L benzocaine solutions. The specific sampling site can be referenced in Fig. S1. After the removal of the skin, muscles labeled as M1, and M2 preserved under varying conditions: M1 was stored at room temperature, fixed with environmentally friendly GD fixing solution (G1111, Servicebio, Wuhan, China), and stored at −20 °C and −80 °C for subsequent experimental analysis. Upon completion of the experiment, the remaining fish were anesthetized and euthanized, and samples were collected and frozen for preservation.

### Histological observation and immunofluorescence

Following prior studies [10], the muscle tissue slices were prepared, stained, and imaged by Chengdu Lilai Biotechnology Co., Ltd. (Chengdu, China). Subsequently, an immunofluorescence assay was performed to detect levels of MYHC and FoxO3a utilizing primary antibodies (MF20, 1:5, DSHB, Iowa, USA; and FoxO3a, 1:100, ABclonal, Wuhan, China).

### Realtime PCR

The procedure was similar to our earlier investigation [53]. The muscle samples were processed for total RNA isolation using RNAiso Plus (Takara, Dalian, China), followed by assessment of RNA quality and quantity using NanoDrop 2000 (Thermo Fisher Scientific) and agarose gel (1.5%) electrophoresis, respectively. Then the RNA was reverse transcribed into cDNA by the PrimeScript® RT reagent kit (Takara, Dalian, China). 2 × SYBR Green qPCR Mix (Aidlab, Beijing, China) was applied for q-PCR reaction with the internal control gene β-actin and specific primers [54], which were designed in accordance with the sequences of grass carp (supplementary Table S2). The 2^−ΔΔCt^ method was used to assess mRNA relative expression levels.

### Western blot (WB) analysis

We used WB to detect the expression level of P-4EBP1^Thr37/46^, T-TOR, P-S6K1^Ser398^, T-4EBP1, P-TOR^Ser2448^, T-S6K1, MyoD, MyoG, F-Box protein 32 (MAFbx), muscle-specific RING finger protein 1 (MuRF1), FoxO1a, and FoxO3a proteins (see Table S3 for detailed information and dilution ratio). The protein extraction steps were as follows: Take 50 mg of gill samples from the −80 °C refrigerator and place them in a 2-mL EP tube prepared and added in advance according to RIPA:PMSF (87:1). Vortexed them for 5 s and placed them on ice. After ultrasonic crushing for 45 s (ultrasonic crushing for 5 s, interval of 10 s, power intensity of 50%), it was centrifuged at 4 °C and 6,000 × *g* for 15 min. Take 100 μL of the supernatant and divide it into 200-μL centrifuge tubes. The bicinchoninic acid (BCA) (P0010, Beyotime, Shanghai, China) method was used to create a concentration absorbance standard curve. The ratio of BCA working solution was A solution:B solution = 50:1, and 18 μL of physiological saline + 2 μL of sample to be tested + 200 μL of BCA working solution was added to the measuring well in the enzyme-linked plate. After incubating at 37 °C for 30 min, read the absorbance value at a wavelength of 562 nm using a full-wavelength enzyme-linked immunosorbent assay. Determined the concentration of the sample from the standard curve (minimum protein concentration > 3 μg/μL; if lower than this concentration, the sample needs to be remade). Dilute the sample with the protein concentration reference value (≥ 10 μg/μL or higher) with a lysis mixture to this concentration. The specific method was to take a certain volume (VO) of the protein sample (90 μL) and calculate the volume (V) of the sample with a concentration higher than the reference value. VO − V was the volume of the required lysis mixture. Note: ensured that the total protein concentration in the homogenized tissue of each treatment was consistent. Mixed the sample or diluted sample with 1/5 volume of 5× sampling buffer (P0015L, Beyotime, Shanghai, China), mixed it in a vortex for 5 s, placed it on a PCR machine, denatured it at 96 °C for 8 min, stored it in an −80 °C refrigerator (about 1 month). The experimental method of the subsequent WB is based on previous research in our laboratory [8]. Table S3 lists the antibody information used in WB analysis.

### Statistical analysis

The data are expressed as mean ± standard deviation. In IBM SPSS Statistics 25 (IBM Corp., Armon, NY, USA), the normal distribution test was performed, and the one-Way ANOVA, Duncan's multiple comparison method and independent sample* t*-test were used to analyze the differences between groups to evaluate the statistical differences between treatments (*P* < 0.05). The graphical abstract was made on BioRender.com. The bar chart was created by GraphPad 8.0 Software.

## Result

### Effects of OTA-exposed on CV in GCPMs and natural product screening

**Fig. 1:**
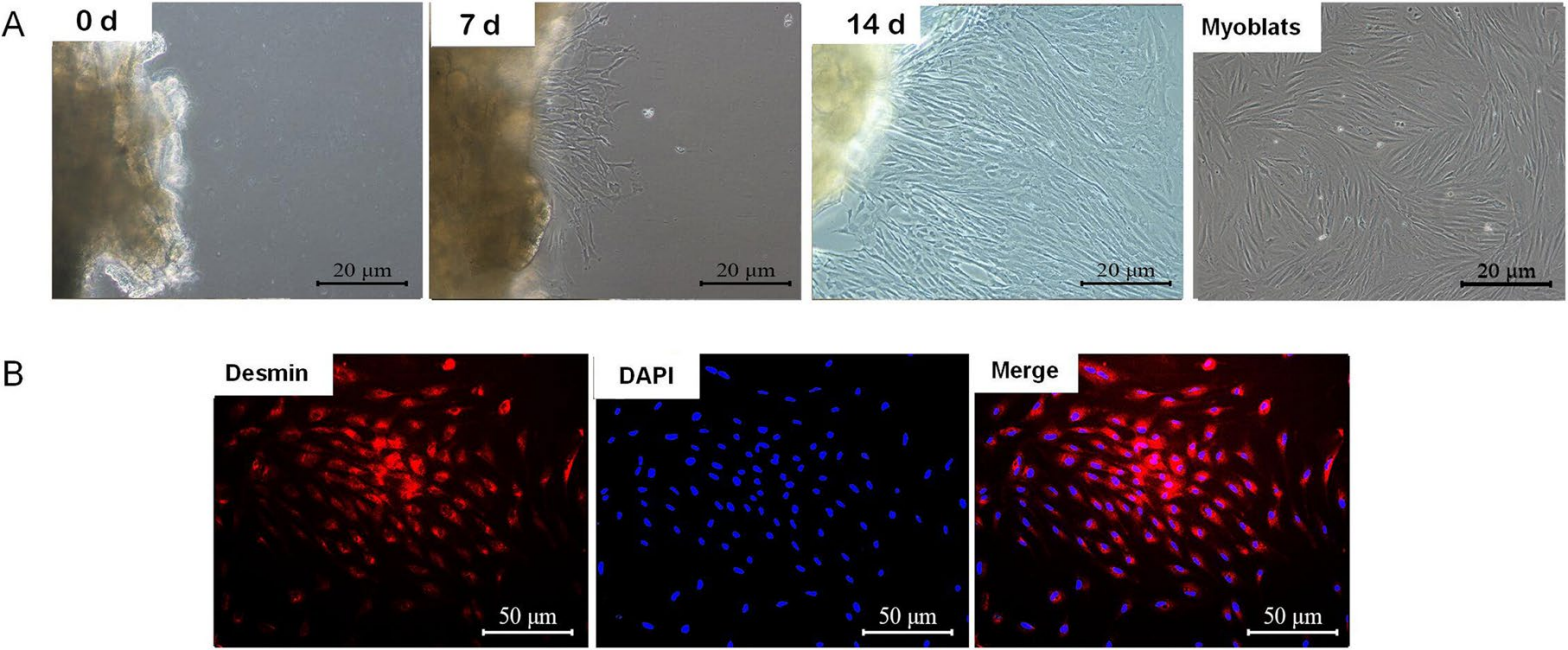
Isolation, culture, and identification of primary myoblasts from grass carp and determination of OTA and Cur dose. **A** Myoblast tissue block was cultured for different day and purified myoblast, × 200; **B** Myoblasts were identified by Desmin immunofluorescence staining, × 400

**Fig. 2:**
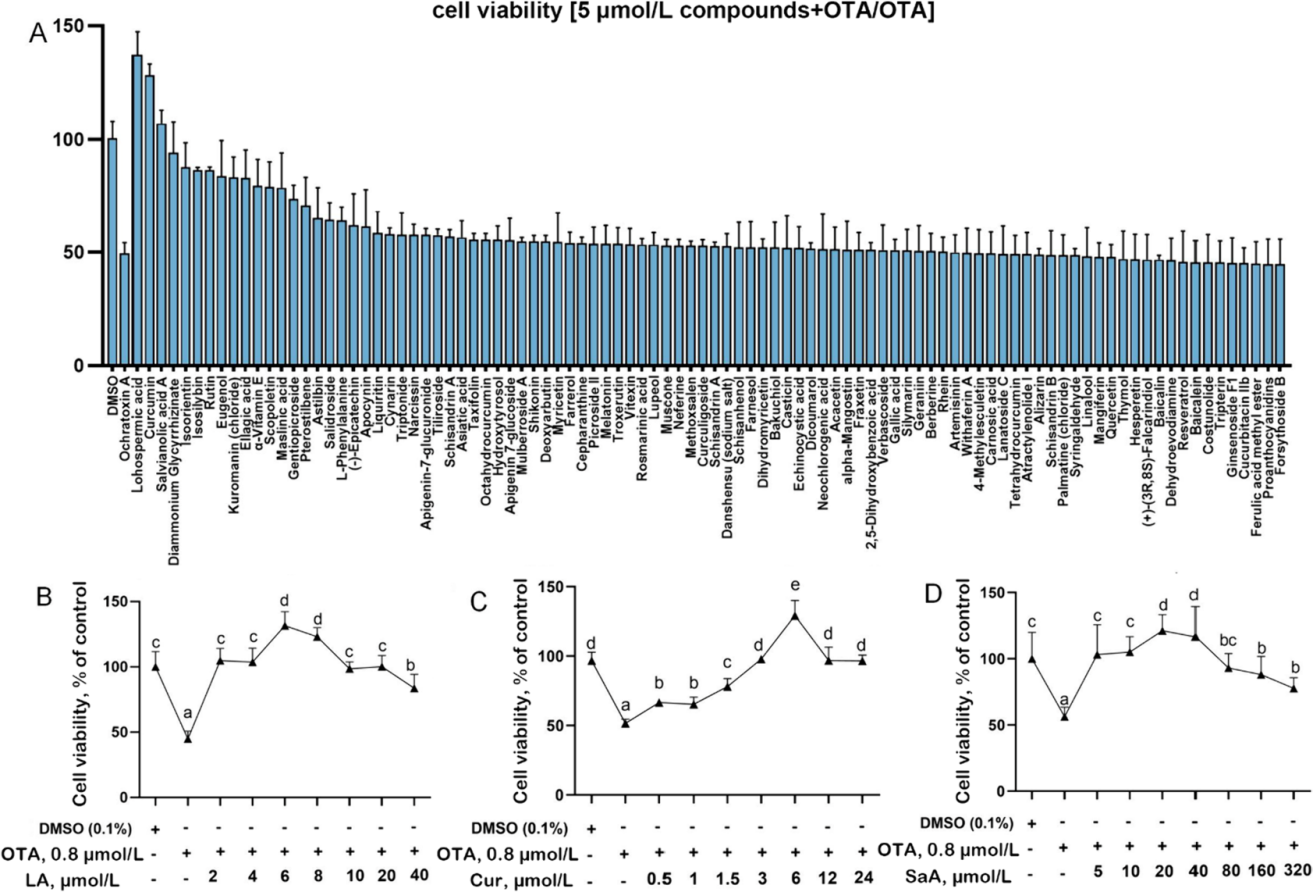
Screening of natural products to alleviate OTA inhibition of cell proliferation. **A** Anti-OTA inhibits cell activity of natural product. Grass carp myoblasts were treated with OTA (0.8 μmol/L) in the absence or presence of the indicated natural product compounds (5 μmol/L). Cell viability was assayed using the CCK-8 kit, *n* = 3. **B–D** CCK8 determination of the optimal concentration of lithospermic acid (LA), curcumin (Cur) and salvianolic acid A (SaA) to alleviate OTA inhibition in cell viability, *n* = 6. Values are presented as mean ± SD. ^a^^–^^e^Different letters indicate significant differences (one-way ANOVAs, *P* < 0.05)

The culture progression of our grass carp GCPMs was visually represented in Fig. 1A, showcasing the growth of GCPMs starting on the 7^th^ d and reaching a range of 60% to 80% by the 14^th^ d. The purified GCPMs were fibrous (Fig. 1A). The purified cells were identified as GCPMs by Desmin immunofluorescence staining (Fig. 1B). The growth curve, based on cell count results, was plotted with cell number (cell number/mL) as the ordinate and time as the abscissa, revealing a logarithmic phase from the 2^nd^ to the 5^th^ d (Fig. S2B). Additionally, the concentration and time gradient tests of OTA on GCPMs were presented in Fig. S2A, respectively. Following exposure to 0.6 μmol/L OTA for 48 and 72 h, a significant decrease in cell viability (CV) was observed in GCPMs (*P* < 0.05). Additionally, the LDH content of GCPMs was significantly increased after treatment with 0.2 and 0.4 μmol/L OTA for 48 and 72 h, respectively (*P* < 0.05) (Fig. S2C and D). The half-maximal inhibitory concentration (IC50) of OTA for 48 h OTA processing was 0.8824 μmol/L (Fig. S3). Consequently, the concentration of 0.8 μmol/L OTA exposed for 48 h was selected for further experimentation. In order to identify potential substances that may mitigate the inhibitory effects of OTA on cell growth, a small library of 96 compounds from MedChemExpress (MCE) was screened. When the natural product compounds lithospermic acid (LA), curcumin (Cur), and salvianolic acid A (SaA) were combined with OTA at concentrations of 2.5, 5, and 10 μmol/L to treat GCPMs, they were found to significantly enhance cell proliferation, surpassing the control group level (*P* < 0.05) (Fig. 2A, S4 and Table S5). For instance, treatment with 5 μmol/L NPL resulted in an increase in cell viability from 49.11% to 136.71% in the LA, Cur, and SaA groups. Subsequent experiments demonstrated that varying concentrations of LA, Cur, and SaA in combination with OTA-exposed GCPMs had differing effects on cell proliferation, with optimal concentrations identified as 6 μmol/L, 6 μmol/L, and 20 μmol/L, respectively (Fig. 2B–D). Due to its low concentration, broad efficacy, and cost-effectiveness, Cur was selected for further experimentation.

### Effect of Cur on differentiation of GCPMs exposed to OTA in vitro and in vivo

**Fig. 3:**
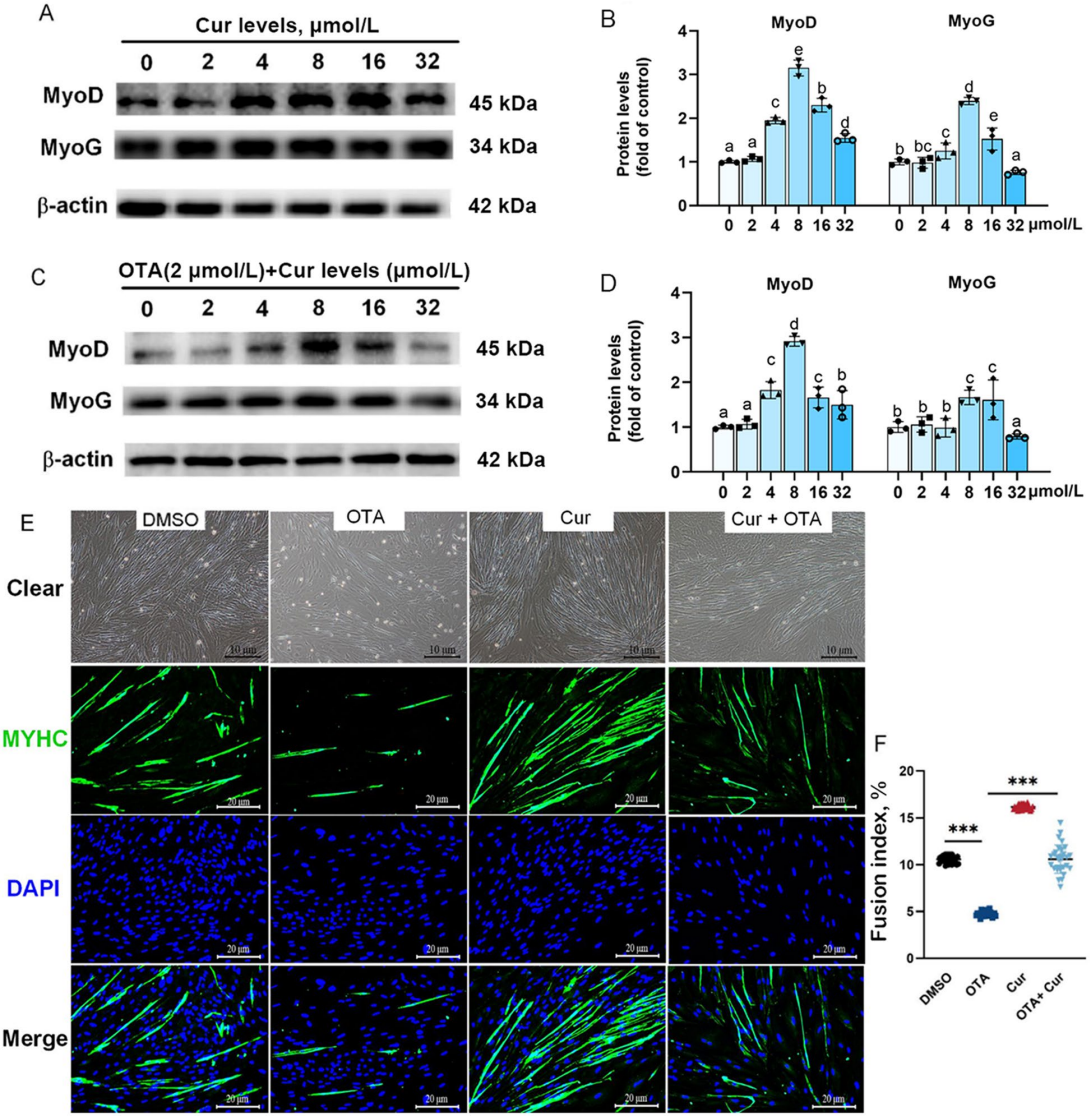
Cur alleviated grass carp primary myoblasts (GCPMs) differentiation change. **A–B** Dose determination of Cur promoted the protein expression of MyoD and MyoG, *n* = 3. **C**–**D** Cur alleviated the inhibition of protein expression of MyoD and MyoG caused by OTA exposure, *n* = 3. **E**–**F** Morphology of myoblasts differentiating into myotubes, × 200. **E** MYHC immunofluorescence staining identifies myotubes and counts fusion index, *n* = 3, × 400. For all quantifications, data (mean ± SD) were from the indicated number of independent experiments. ^a^^–^^e^Different letters indicate significant differences (*P* < 0.05). Four treatments were tested by independent-sample *t*-test, *P* < 0.001 (***) vs. the control group

The results depicted in Fig. S2 and S3 indicate that the expression of MYHC and MyoG proteins during in myoblast differentiation were significantly decreased by 2 μmol/L OTA triggering compared with the 0.1% DMSO group (*P* < 0.05). The protein expressions of MyoD and MyoG were elevated to their peak levels following exposure to 8 μmol/L Cur (Fig. S2E and F). The supplementation of 8 μmol/L Cur effectively inhibited the impaired effect of OTA (2 μmol/L) on MyoD and MyoG protein expressions and promoted myoblasts to differentiate into myotubes (*P* < 0.05) (Fig. 3A–B). The morphology of myoblasts differentiating into myotubes (Fig. 3C and D) and immunofluorescence staining of MYHC also exhibited the same result (Fig. 3E and F). Compared with the OTA group, the number of mature muscle fibers in the Cur + OTA group significantly increased, and the addition of the Cur significantly alleviated the decline of the myotube fusion induced by OTA (*P* < 0.001) (Fig. 3E and F).

**Fig. 4:**
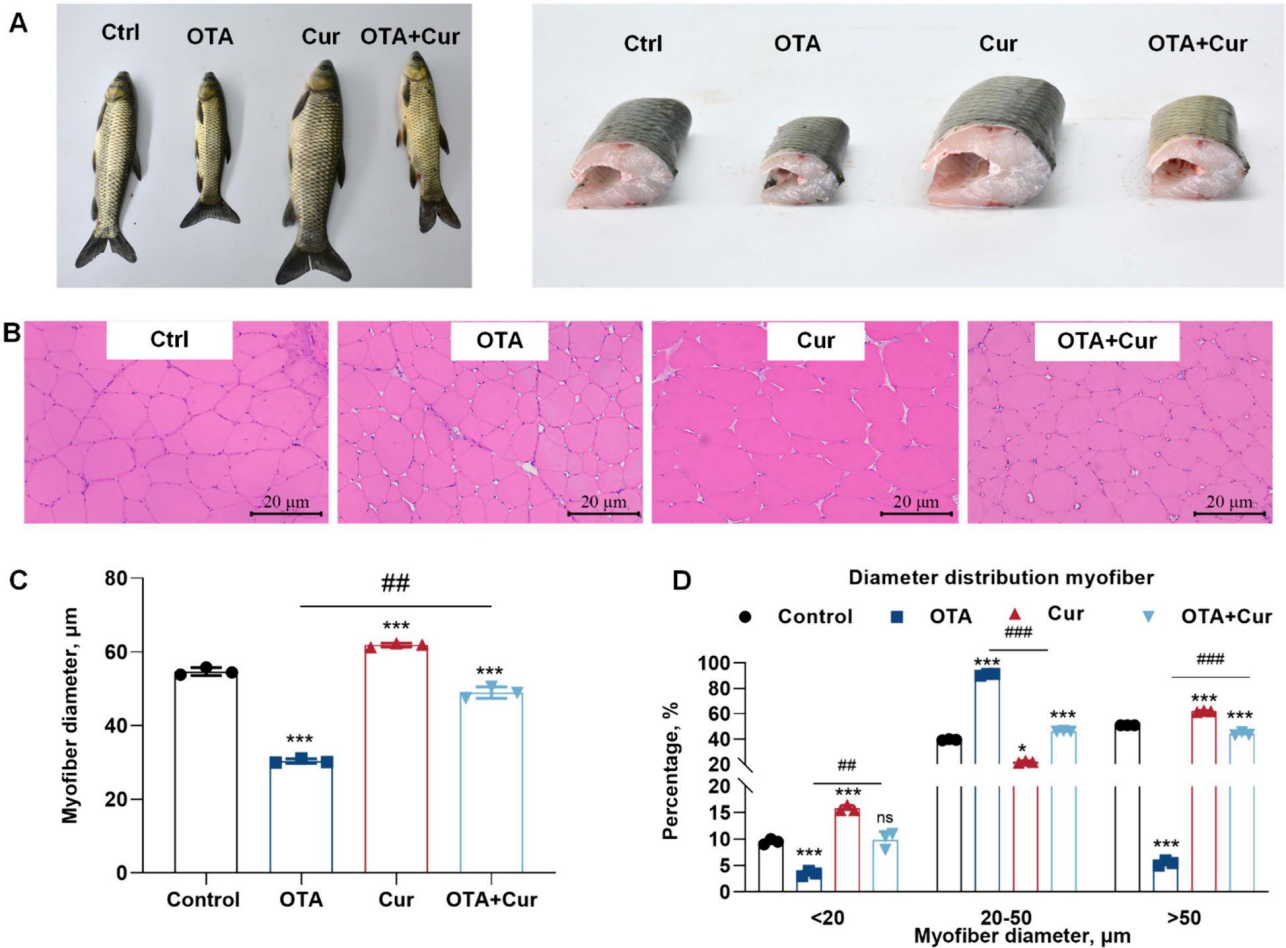
Cur reversed OTA-impaired grass carp's growth performance and morphological characteristics changes of myofibers. **A–B** The muscle cross-section was measured by hematoxylin–eosin staining, × 200; **C** Muscle fiber diameter; **D** Distribution frequency of muscle fiber diameter (%). For all quantifications, data (mean ± SD) were from the indicated number of independent experiments. Independent-Sample *t*-test, the no horizontal line indicates *P* < 0.05 (*), *P* < 0.01 (**), *P* < 0.001 (***) vs. the control group; The horizontal line indicates *P* < 0.05 (^#^), *P* < 0.01 (^##^), *P* < 0.001 (^###^) vs. OTA-exposure group; ns = not significant

**Fig. 5:**
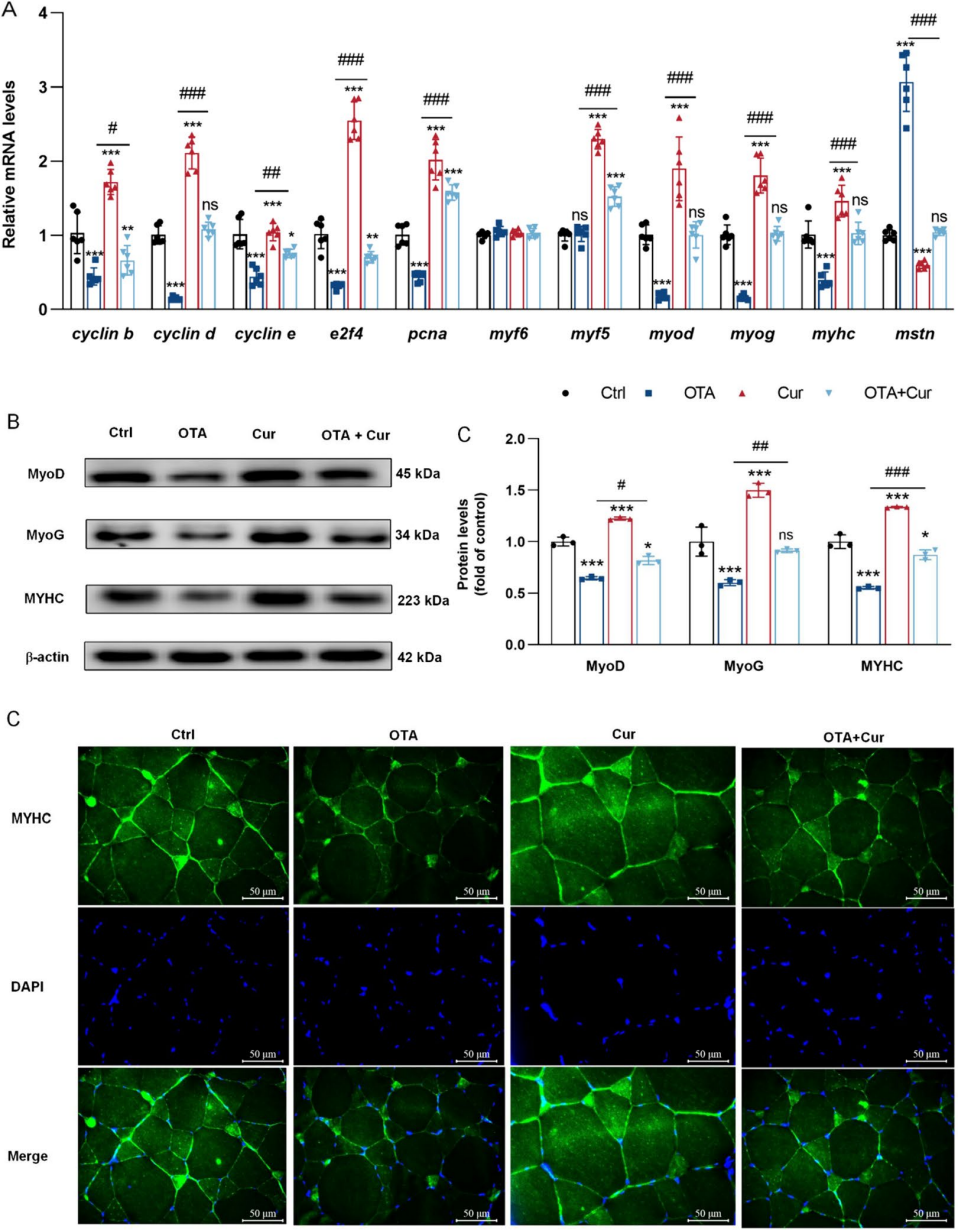
Cur alleviated OTA-inhibited grass carp's muscle fiber development. **A** Regulation of gene expression related to protein synthesis and degradation, *n* = 6. **B** Regulation of protein expression related to protein synthesis and degradation, *n* = 3. **C** MYHC immunofluorescence staining, × 400. For all quantifications, data (mean ± SD) were from the indicated number of independent experiments. Independent-sample *t*-test, the no horizontal line indicates *P* < 0.05 (*), *P* < 0.01(**), *P* < 0.001 (***) vs. the control group; The horizontal line indicates *P* < 0.05 (^#^), *P* < 0.01 (^##^), *P* < 0.001 (^###^) vs. OTA-exposure group; ns = not significant

In vivo, as in our previous study [54], Cur increased OTA-suppressed weight gain (WG), specific growth rate (SGR), feed efficiency (FE), feed intake (FI), and food coefficient rate (FCR) compared to the OTA-alone group (*P* < 0.05). Meanwhile, the results presented in Fig. 4A–D indicate a significant decrease in the diameter of muscle fiber in grass carp when exposed to OTA (*P* < 0.001). However, co-treatment with OTA and Cur resulted in a noticeable increase in muscle fiber diameter compared to the OTA group (*P* < 0.01). Furthermore, OTA treatment alone led to a significant decrease in the percentages of myofiber diameters of <20 μm and >50 μm compared to the control group (*P* < 0.001). Conversely, co-treatment with Cur + OTA significantly increased the percentages of myofiber diameters <20 μm and >50 μm compared to the OTA group (*P* < 0.01). The opposite pattern was observed for the 20–50 μm myofiber diameter ratio (Fig. 4D). In addition, there was a significant decrease in the mRNA expression levels of *cyclins (b, d, e), e2f4, pcna,* *myf5, myod, myog,* and *myhc*, as well as in the protein expressions of MyoD, MyoG, and MYHC, and an increase in MSTN expression following OTA treatment compared to the control group (*P* < 0.01 or *P* < 0.001) (Fig. 5A and B). Conversely, co-treatment with Cur and OTA resulted in a significant increase in the gene expressions of *myf5, myod, myog,* and *myhc* as well as in the protein expressions of MyoD, MyoG, and MYHC and a decrease in *mstn* gene expressions compared to OTA-treatment group (*P* < 0.01 or *P* < 0.001). Furthermore, as depicted in Fig. 5C, Cur treatment reversed the effects of OTA compared to the OTA group alone in MYHC immunofluorescence intensity.

### Cur promoted OTA-inhibited GCPMs and muscle protein deposition in vitro and in vivo

**Fig. 6:**
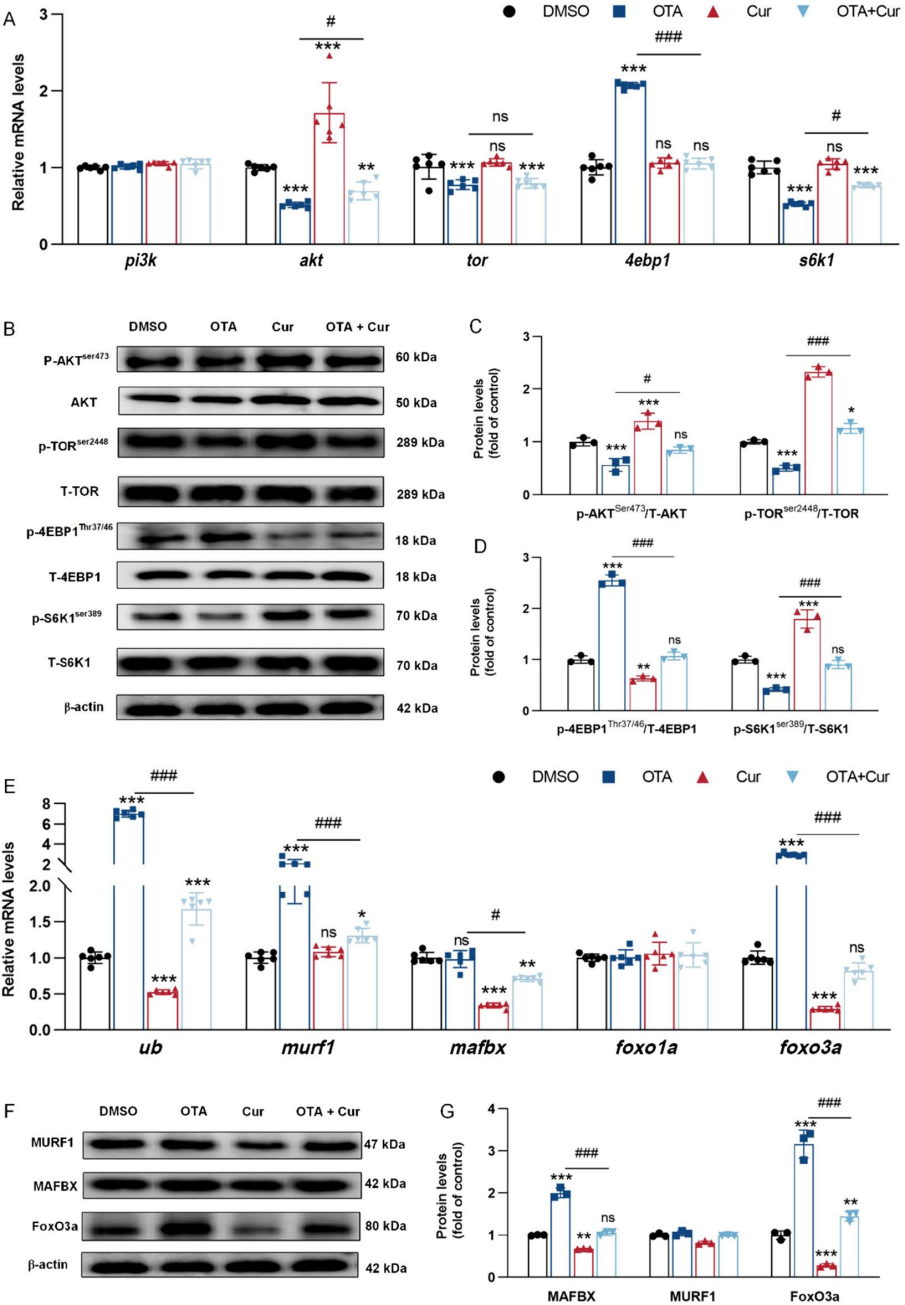
Cur alleviated OTA-impaired grass carp myoblasts protein deposition. **A** Regulation of gene expression related to protein synthesis, *n* = 6. **B–D** Regulation of protein expression related to protein synthesis and degradation, *n* = 3. **E** Regulation of gene expression related to protein degradation, *n* = 6. **F–G** Regulation of protein expression related to protein degradation, *n* = 3. For all quantifications, data (mean ± SD) were from the indicated number of independent experiments. Independent-sample *t*-test, the no horizontal line indicates *P* < 0.05 (*), *P* < 0.01(**), *P* < 0.001 (***) vs. the control group; The horizontal line indicates *P* < 0.05 (^#^), *P* < 0.01 (^##^), *P* < 0.001 (^###^) vs. OTA-exposure group; ns = not significant

**Fig. 7:**
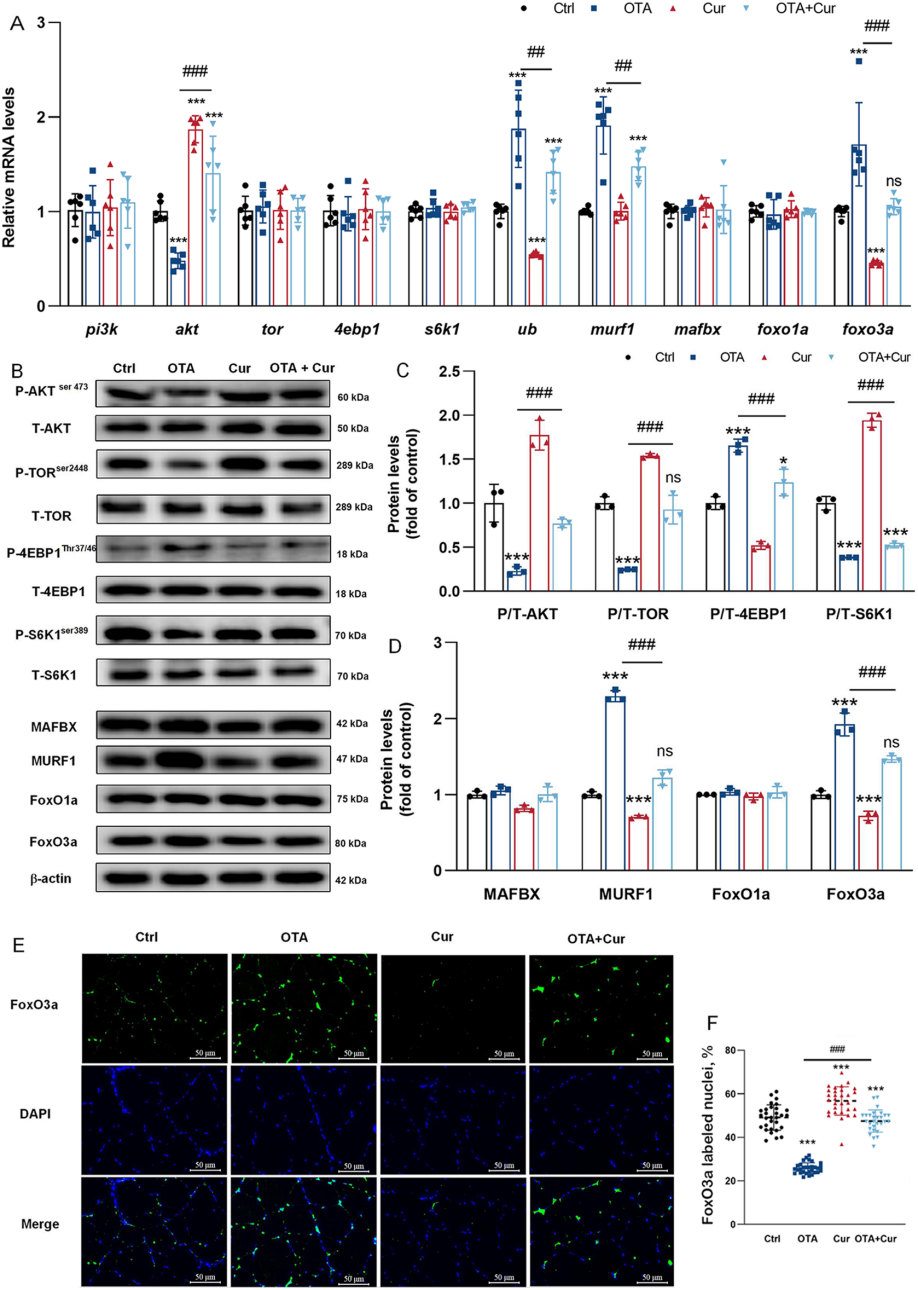
Cur alleviated OTA-impaired grass carp muscle protein deposition. **A–B** Regulation of gene (*n* = 6) and protein (*n* = 3) expression related to protein synthesis and degradation. **C** FoxO3a immunofluorescence staining and statistical results, × 400, *n* = 3. For all quantifications, data (mean ± SD) were from the indicated number of independent experiments. Independent-sample *t*-test, the no horizontal line indicates *P* < 0.05 (*), *P* < 0.01(**), *P* < 0.001 (***) vs. the control group; The horizontal line indicates *P* < 0.05 (^#^), *P* < 0.01 (^##^), *P* < 0.001 (^###^) vs. OTA-exposure group; ns = not significant

Protein deposition is intricately linked to the processes of protein synthesis and degradation [55, 56]. As shown in Fig. 6A and E, the gene expressions of *AKT, TOR* and ribosomal protein S6 kinase 1 (*S6K1*) were significantly decreased in the grass carp myoblasts induced by OTA (*P* < 0.05), whereas the eIF4E-binding protein 1 (*4EBP1*)*, murf1, foxo3a, *and* ub* mRNAs were significantly increased with OTA-treated cells in vitro (*P* < 0.001), compared with the control group. Additionally, co-treatment with Cur and OTA led to a significantly increased the *akt *and* s6k1* and decreased the *4ebp1*, *murf1, foxo3a, *and* ub* mRNAs (*P* < 0.01 or *P* < 0.001) (Fig. 6A and E), compared with OTA-exposure group in vitro. However, the addition of OTA and Cur alone or in conjunction with other treatments did not affect the gene expressions of *pi3k*, and *foxo1a* (*P* > 0.05) in vitro. In vivo experiment, the effect on the expression of other proteins was consistent with that in vitro experiment, except that there were no effects on the *tor, 4ebp1*, and *s6k1* mRNA abundances (Fig. 7A). Furthermore, both in vivo and in vitro experiments (refer to Figs. 6B–D and 7B–C) demonstrated a significant decrease in phosphorylation-4EBP1 on Thr37/46 (P-4EBP1^Thr37/46^)/total-4EBP1(T-4EBP1) protein abundances, while the phosphorylation-TOR on Ser2448 (P-TOR^Ser2448^) total-TOR (T-TOR), phosphorylation-AKT on Ser473 (P-AKT^Ser473^)/total-AKT(T-AKT) and phosphorylation-S6K1 on Ser398 (P-S6K1^Ser398^)/total-S6K1(T-S6K1) were significantly increased with OTA + Cur exposure (*P* < 0.01 or *P* < 0.001). In contrast, the protein expressions of MURF1 and FoxO3a exhibited a significant increase following exposure to OTA, while they decreased significantly (*P* < 0.001) with Cur-treatment (Fig. 6F–G and 7B and D). Conversely, neither OTA, Cur, nor their combined exposure had a discernible impact on the protein expression of FoxO1a (*P* > 0.05). Immunofluorescence results of FoxO3a displayed that the nucleation rate of FoxO3a was significantly reduced in the OTA and Cur co-treatment groups compared with the OTA alone supplement group (*P* < 0.001) (Fig. 7E and F).

### Cur alleviated OTA inhibited protein deposition through the AKT/FoxO3a signaling pathway in vitro

**Fig. 8:**
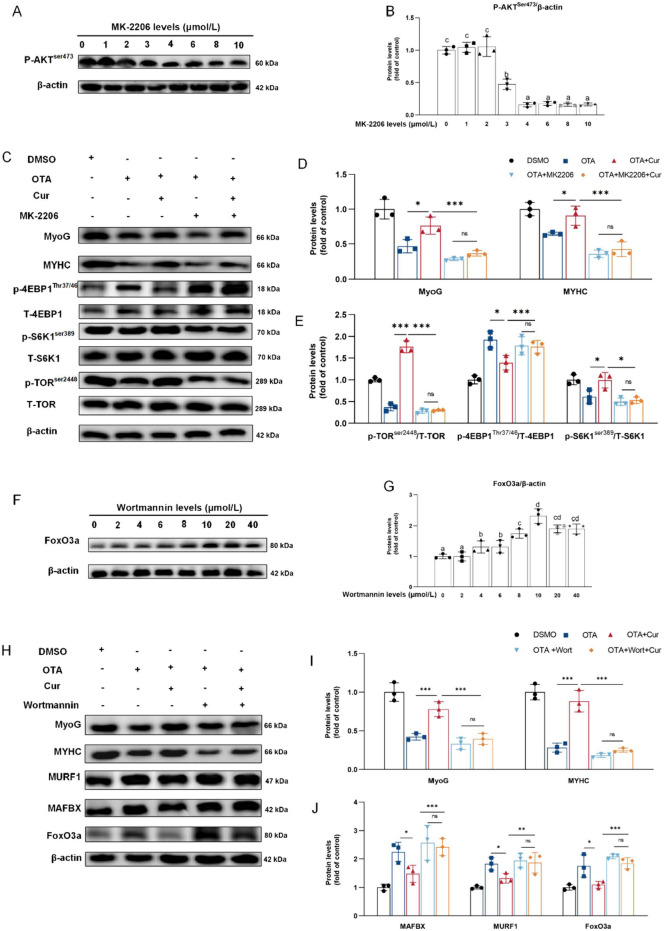
Cur alleviated OTA induced protein deposition reduction in myotube cells through the AKT/FoxO3a signaling pathway. **A–B** The effect of different doses of MK-2206 (AKT inhibitor) on myotube phosphorylated-AKT^ser473^ protein expression (*n* = 3). **C–E** With OTA exposure conditions, Cur enhanced protein synthesis in myotube cells through the AKT/TOR pathway, the WB results of MyoG, MYHC, p-TOR, p-4EBP1, and p-S6K1, *n* = 3. **F–G** The effect of different doses of wortmannin (FoxO3a agonist) on myotube FoxO3a protein expression (*n* = 3). **H–J** With OTA exposure conditions, Cur decreased protein degradation in myotube cells through the FoxO3a pathway, the WB results of MyoG, MYHC, MURF1, MAFBX, and FoxO3a, *n* = 3. For all quantifications, data (mean ± SD) were from the indicated number of independent experiments. Different superscripts indicate significant differences (one-way ANOVAs, *P* < 0.05). Independent-sample *t*-test, *P* > 0.05 (ns), *P* < 0.05 (*), *P* < 0.01(**), *P* < 0.001 (***)

The determination of optimal dosage of the AKT inhibitor MK-2206 in primary myotube cells (GCPMTs) was illustrated in Fig. 8A and B. Following the administration of varying concentrations of MK-2206, a notable decrease in p-AKT protein levels was observed at 3 μmol/L, with no significant difference noted at dosages ranging from 4–10 μmol/L (*P* > 0.05). Consequently, a dosage of 4 μmol/L MK-2206 was chosen for subsequent experiments. Subsequent to OTA exposure, the addition of MK-2206 significantly affected the expression of MyoG, MYHC, P-TOR, and P-S6K1 proteins in GCPMTs (Fig. 8C–E). Compared with OTA alone exposure group, the protein expressions of MyoG, MYHC, P-TOR, and P-S6K1 were significantly increased in the OTA + Cur co-addition group (*P* < 0.001). The protein expressions of MyoG, MYHC, P-TOR, P-S6K1 and P-4EBP1 in OTA + MK-2206 + Cur group were not significantly different from those in OTA + MK-2206 + Cur group (*P* > 0.05). The dose determination of FoxO3a activator Wortmannin (Wort) was shown in Fig. 8F and G. The 4 μmol/L Wort could decrease the protein level of FoxO3a significantly (*P* < 0.05). The difference was not significant in the 10–40 μmol/L Wort (*P* > 0.05), so 10 μmol/L Wort was selected for follow-up experiments. As a result of OTA exposure, the addition of Wort significantly affected the protein expression of MURF1, MAFBX and FoxO3a in GCPMTs (Fig. 8H–J). Compared with OTA alone exposure group, the protein expressions of MURF1, MAFBX and FoxO3a were significantly decreased in the OTA + Cur co-addition group (*P* < 0.05 or *P* < 0.001). The protein expressions of MURF1, MAFBX and FoxO3a in OTA + Wort + Cur group were not significantly different from those in OTA + Wort group (*P* > 0.05).

## Discussion

### Establishment of GCPMs and screening of natural products to alleviate OTA cytotoxicity

Primary fish cells are a great advantage over cell lines because they mimic in vivo conditions better than fish cell lines, which are also important for studying toxicology [57]. Currently, no relevant research has been conducted on the tissue culture method of GCPMs. In this study, primary myoblasts were isolated and cultured from grass carp muscle via the tissue block culture method and identified by Desmin and MYHC immunofluorescence staining [58]. The differential adhesion method to purifying cells was consistent with that in zebrafish and rainbow trout primary myoblasts, all of which were long fusiform fibroblasts [59].

OTA, one of the most prevalent mycotoxins in nature, has raised concerns due to its diverse toxic effects on animals [60]. NPL is commonly used as an effective tool for screening out mitigating substances against the toxicity of target substances [61–63]. Despite the lack of existing research on the alleviation of OTA toxicity through NPL screening on cells, our study utilized NPL screening to identify three initial substances (6 μmol/L LA, 6 μmol/L Cur and 20 μmol/L SaA) that significantly increased cell viability compared to the OTA-treated group alone, with increases of cell viability (CV) by 87.6%, 78.63%, and 57.47% respectively. LA and SaA belong to the salvianolic acid family, which was water-soluble polyphenolic compounds with anti-inflammatory and antioxidant effects [64]. Recently, there were no studies on LA and SaA alleviating mycotoxin toxicity, but SaA could alleviate the toxicity of arsenic trioxide (ATO) induced cardiac toxicity in rats and the toxicity of rat ventricular myocytes (ARVMs) [65]. However, as a naturally occurring polyphenol derived from turmeric, Cur demonstrates various pharmacological effects, including anti-inflammatory and antioxidant properties [66]. Numerous studies have provided evidence has shown the protective roles of Cur against OTA-induced PK15 cytotoxicity [67], nephrotoxicity induction in rats and ducks [41], hepatotoxicity in rat [68], liver oxidative injury [40], and intestinal barrier dysfunction [39]. These findings suggest that curcumin may be a promising candidate for mitigating the toxicity of mycotoxins. Additionally, curcumin was found to be the most cost-effective option among LA, curcumin, and SaA, leading to its selection for further experiments.

### Cur alleviated OTA-induced inhibition of myoblast proliferation in vitro and growth performance in vivo

The growth of fish is primarily dependent on muscle growth, which is largely attributed to the development of muscle fibers within the muscle [69]. In order to ascertain the inhibitory effects of OTA on muscle growth, the presence of OTA residues in grass carp muscles was analyzed, with levels of 8.34 ± 0.10 μg/kg detected, while the group supplemented with Cur did not show any OTA residues (Table 1). Additionally, it remains unclear whether the inhibition of muscle growth is related to myoblast development impairment. Consistently, compared with OTA alone treatment group, we found that Cur and OTA co-treated fish improved growth performance mainly by increasing percentage weight gain and final weight, as well as gene expression of cell cycle-related proteins, cyclin (B, D, E), E2F4, and PCNA in vivo. This is consistent with studies on ducks [40], which indicated Cur could improve growth performance inhibition caused by OTA. Above data suggested that Cur could alleviate the OTA-inhibited myoblast proliferation in vitro and promote growth in vivo.

**Table 1:** The ochratoxin A residual of intestine of grass carp muscle

OTA content, μg/kg tissue	Groups			
	0	OTA	Cur	OTA + Cur
Muscles	n.d.^a^	8.34 ± 0.10^***^	n.d.^a^	n.d.^a^

^a^ Indicates not detected. Using independent sample *t*-test, the data is represented by mean ± standard deviation (*n* = 6)

^***^ Represent significant differences between the OTA + Cur group and OTA (*P* < 0.001)

### Cur alleviated OTA-induced inhibition of myoblast differentiation and fusion in vitro and in vivo

The differentiation and fusion of myoblasts play a crucial role in muscle hypertrophy and growth, as indicated by previous studies [46]. As muscle fibers mature, they are categorized into three diameter classes (< 20 µm, 20–50 µm or > 50 µm) [70]. Among them, muscle fiber hyperplasia and hypertrophy can be reflected by the frequency of muscle fibers with diameters < 20 μm and > 50 μm, respectively [71]. Our findings demonstrate a significant decrease in the frequency of muscle fibers with diameters < 20 μm and > 50 μm in the group treated with OTA, but this effect was reversed by Cur. Furthermore, myoblast differentiation is regulated by myoblasts regulatory factors (MRFs) [72]. Specifically, MyoD and MyoG are differentiation markers for myoblasts, and their expression indicates whether myotube cells have matured [73]. The results of our study demonstrated that the addition of OTA had a significant inhibitory effect on the differentiation of myoblasts, but with the increased in the dose of Cur, the protein expression of MyoD and MyoG reached the highest level at 8 μmol/L and significantly increased the fusion index of myotubes. These indicated that Cur significantly improved the differentiation of myoblasts inhibited by OTA. Findings of the present vivo study also showed that the co-addition of Cur and OTA significantly increased gene and the protein expressions of MyoD, MyoG and MYHC compared to the OTA treatment group. Therefore, Cur could potentially mitigate the hindrance of muscle fiber growth caused by OTA through its role in promoting cell differentiation, which is linked to the upregulation of the Myogenic Regulatory Factors (MRFs) family.

### Cur alleviated OTA-induced inhibition of protein deposition in vitro and in vivo

The fusion of myoblasts is intricately linked to muscle protein deposition, a process primarily influenced by the balance of protein synthesis and degradation [55, 56]. The AKT/TOR signaling pathway plays a critical role in regulating protein synthesis with downstream S6K1 and 4EBP1 activation [49, 74]. As a result of our research, we found that Cur + OTA co-treatment could reduce the protein expression of P-4EBP1^Thr37/46^/T-4EBP1, and increase the P-AKT^Ser473^/T-AKT, P-S6K1^Ser398^/T-S6K1, P-TOR^Ser2448^/T-TOR compared to treatment with OTA alone. Interestingly, all groups exhibited maintained *tor* and *4ebp1* mRNA expressions, suggesting gene regulation may occur at various levels such as transcriptional, post-transcriptional, translational, and post-translational [75]. This might be because the treatment with OTA, Cur or OTA + Cur tends to impact its phosphorylation expression but not its transcription expression. However, this hypothesis needs further verification. Subsequent studies revealed that the addition of Cur did not enhance the expression of P-AKT^Ser473^/T-AKT, P-S6K1^Ser398^/T-S6K1, P-TOR^Ser2448^/T-TOR and inhibited the expression of P-4EBP1^Thr37/46^/T-4EBP1 protein compared with OTA + MK-2206 group. These findings indicate that Cur may potentially attenuate the inhibitory effects of OTA on protein synthesis through modulation of the AKT/TOR signaling pathway. Although there is currently a lack of research specifically examining the effects of Curcumin on OTA-induced reduction in muscle protein synthesis, previous studies in rats have demonstrated its ability to mitigate muscle atrophy via the AKT/TOR pathway [76]. Additionally, in vitro studies have shown that Cu can alleviate lipopolysaccharide-induced muscle atrophy through the PI3K/AKT signaling pathway [77]. Studies on other toxins also showed that Cur could alleviate nephrotoxicity caused by Fumonisin B1 through the AKT/TOR signaling pathway [78]. Thus, the AKT/TOR pathway was the typical signaling pathway by which Cur could alleviate protein synthesis inhibition caused by OTA.

Furthermore, the ubiquitin/proteasomal-dependent pathway serves as the primary mechanism for protein degradation [79]. This pathway is intricately regulated by the FoxO signaling pathway and related effector molecules MURF1 and MAFBX [80, 81]. Our in vivo and in vitro studies revealed that treatment with Cur in the presence of OTA exposure effectively suppressed the mRNA and protein expressions of MAFBX and FoxO3a. Interestingly, the addition of the FoxO3a activator wortmannin did not lead to a decrease in the expression levels of MURF1, MAFBX, and FoxO3a. These findings suggest that Cur mitigates OTA-induced protein degradation through a FoxO3a-dependent mechanism. This finding aligns with previous research conducted in mice, where administration of LPS resulted in muscle atrophy in rats. Conversely, supplementation with Cur significantly attenuated muscle protein degradation [82]. Dietary supplementation of Cur inhibited the expression of MAFBX and MURF1 when dexamethasone induced muscle atrophy in mice [83]. However, study in neuroblastoma (NB) demonstrated that Cur supplementation can inhibit cell proliferation by promoting the expression of Foxo3a and leading to cell apoptosis This may be related to the difference between tumor cells and normal cells [84]. This may be related to the difference between tumor cells and normal cells. Interestingly, OTA and Cur had an impact on FoxO3a mRNA or protein levels in vivo or in vitro, but not on FoxO1a. These findings suggest that Cur's inhibition of protein degradation induced by OTA may be attributed, at least in part, to the suppression of FoxO3a, rather than regulation through FoxO1a. In a murine metabolic syndrome (MetS) rat heart, it has been demonstrated that the gene expression of FoxO3a was notably up-regulated rather than FoxO1a [43]. Our findings diverged from those of a study on aluminum phosphate (ALP) induced hepatotoxicity in mice, which attributed the condition to the downregulation of FoxO1a and FoxO3a. The introduction of Cur was found to mitigate the suppression of FoxO1a caused by ALP, rather than FoxO3a [85]. These discrepancies may be attributed to variations in toxins, species, and organs studied, warranting further investigation for clarification.

## Conclusion

The systematic NPL screen was applied in GCPMS and Cur was selected for the best candidate to alleviate muscle developmental toxicity induced by OTA with the exhibition of increasing the diameter of muscle fibers inhibited by OTA to a frequency of < 20 and > 50 μm muscle fiber distribution. This effect is believed to be mediated by the promotion of myoblast proliferation and fusion through the activation of AKT/TOR signaling and the inhibition of FoxO3a. This study is the first to screen natural products that alleviate the toxic effects of OTA in vitro, and to verify in vitro and in vivo that Cur can alleviate the muscle development of grass carp inhibited by OTA. This study establishes a natural product screening method targeting myoblasts to reduce the speed, feasibility, and effectiveness of mycotoxin toxicity.

### Supplementary Information

**Additional file 1: Table S1.** List of reagent and assay kits used in experimental diet and biochemical parameters analysis. **Table S2.** Real-time PCR primer sequences. **Table S3.** Target proteins, dilution factor, antibody Cat. No. and antibody source of proteins selected for analyzing by western blotting. **Table S4.** Ingredients and nutritional composition of the basal diet. **Table S5.** Raw data of mitigation screening results. **Fig. S1.** Schematic diagram of muscle sampling sites for grass carp. **Fig. S2.** Determination of the dose of OTA inhibiting the proliferation and differentiation of primary myoblasts in grass carp. **Fig. S3.** IC50 results of OTA cytotoxicity to grass carp primary myoblast were calculated by Graphpad 8. **Fig. S4. **Anti-OTA inhibits cell viability of natural product.

## Data Availability

The datasets are included in this article and available from the corresponding author on reasonable request.

## Abbreviations

AKT: Protein kinase B
BCA: Bicinchoninic acid
bEGF: Basic epidermal growth factor
bFGF: Basic fibroblast growth factor
CCK-8: Cell counting kit-8
CP: Cell proliferation
Cur: Curcumin
CV: Cell viability
DAPI: 4',6-Diamidino-2-phenylindole
DMSO: Dimethyl sulfoxide
4EBP1: Eukaryotic initiation factor 4E-binding protein 1
E2F4: E2F transcription factor 4
EP: Eppendorf
FoxO3a: Forkhead box O
GCPM: Grass carp primary myoblast
GCPMT: Grass carp primary myotube cell
IGF1: Insulin-like growth factor 1
LA: Lithospermic acid
MAFbx: F-Box protein 32
MuRF1: Muscle-specific RING finger protein 1
MYHC: Myosin heavy chain
MyoD: Myoblast determination protein
MyoG: Myogenin
NPL: Natural product library
PBS: Phosphate buffered saline
PCNA: Proliferating cell nuclear antigen
q-PCR: Quantitative polymerase chain reaction
SaA: Salvianolic acid A
S6K1: Ribosomal protein S6 kinase 1
TOR: Target of rapamycin
Ub: Ubiquitin
UPS: Ubiquitin-proteasome system
