# Supplementary material for: Unveiling the emerging role of curcumin to alleviate ochratoxin A-induced muscle toxicity in grass carp (Ctenopharyngodon idella): in vitro and in vivo studies
Source: J Anim Sci Biotechnol. 2024 May 12;15:72. doi: 10.1186/s40104-024-01023-6 (PMC11088780; doi:10.1186/s40104-024-01023-6)
Supplement: Supplementary file 1 — Additional file 1: Table S1. List of reagent and assay kits used in experimental diet and biochemical parameters analysis. Table S2. Real-time PCR primer sequences. Table S3. Target proteins, dilution factor, antibody Cat. No. and antibody source of proteins selected for analyzing by western blotting. Table S4. Ingredients and nutritional composition of the basal diet. Table S5. Raw data of mitigation screening results. Fig. S1. Schematic diagram of muscle sampling sites for grass carp. Fig. S2. Determination of the dose of OTA inhibiting the proliferation and differentiation of primary myoblasts in grass carp. Fig. S3. IC50 results of OTA cytotoxicity to grass carp primary myoblast were calculated by Graphpad 8. Fig. S4. Anti-OTA inhibits cell viability of natural product. [file 40104_2024_1023_MOESM1_ESM.docx]

**Table S1** List of reagent and assay kits used in experimental diet and biochemical parameters analysis

| **Reagent** | **Abbreviation** | **Supplier** | **Catalog No.** |
| --- | --- | --- | --- |
| Ochratoxin A  (purity > 98%) | OTA | Pribolab Pte, Ltd. (Singapore) | MSS1020 |
| horse serum | HS | Gibco (Carlsbad, CA, USA) | 16050-122 |
| M199 medium | M199 | Procell Co., Ltd. (Wuhan, China) | PM150616 |
| Natural product library | NPL | MCE | HY-L021 |
| Curcumin  (purity > 98%) | Cur | Cohoo Bio-tech Co., Ltd. (Guangzhou, China) |  |
| Cell Counting Kit-8 | CCK8 | Shenggong Bio-Engineering Institute (Shanghai, China) | E606335-0500 |
| EdU Assay Kit | EdU | RiboBio Biotechnology Co., Ltd.  (Guangzhou, China) | C10310-1 |
| PI/RNase Staining Kit | PI | BD Biosciences (USA) | 550825 |
| DAPI Staining Kit | DAPI | Beyotime (Shanghai, China) | C1006 |
| SABC Assay Kit | SABC | BOSTER Biological Technology Co., Ltd. (Wuhan, China) | SA1050 |
| Alexa Fluor 488 goat anti-rabbit antibody | secondary antibodies | Beyotime (Shanghai, China) | A0423 |

**Table S2** Real-Time PCR primer sequences

| **Target gene** | **Primer sequence forward (5′ → 3′)** | **Primer sequence reverse (5′ → 3′)** | **Accession number** |
| --- | --- | --- | --- |
| *tor* | TCCCACTTTCCACCAACT | ACACCTCCACCTTCTCCA | [1] |
| *S6k1* | TGGAGGAGGTAATGGACG | ACATAAAGCAGCCTGACG | EF373673 |
| *4ebp1* | GCTGGCTGAGTTTGTGGTTG | CGAGTCGTGCTAAAAAGGGTC | KT757305 |
| *foxo1a* | GCATCTCATAGCCATGCCCT | CACCTCCAAGATGACCGGAG | KP325483 |
| *foxo 3a* | GCTGCGTAGTGATCCCATGATGTC | GGAGACTGTTGGAGATGCTGCTTC | [1] |
| *murf1* | TGTCTATGGACTACAGAGGAA | GGATTTCAAAGGAGGTTCAAG | [1] |
| *mafbx* | CGGACGAGATCTGGTTAGCC | GCTTGCGGATCTGTCTGTCT | [1] |
| *ub* | GCCAAGCGACACCATTGAG | GGATGTTGTAGTCGGACAG | [1] |
| *cyclin B* | TTATTGACTGGCTTGTGC | TTATTGACTGGCTTGTGC | *KX085204* |
| *cyclin D* | TCAGTGACACCGCACGAT | TCAGTGACACCGCACGAT | *KX085205* |
| *cyclin E* | ACTTGGGTCAGGACTACTTTG | ACTTGGGTCAGGACTACTTTG | *KX682385.1* |
| *e2f4* | CAAGTTGGAATTGGAGGAT | CAAGTTGGAATTGGAGGAT | *KX085206* |
| *pcna* | CCTACCGCTGCGACAGAAACCT | TCAAAGACAAGAGCCAAAGAGT | *JX524136* |
| *myog* | AGAGGAGGTTGAAGAAGGTC | GTTCCTGCTGGTTGAGAGA | JQ793897 |
| *myod* | CCCTTGCTTCAACACCAACG | TCTCCTCTCCCTCATGGTGG | GU218462 |
| *myf6* | GAAAATCTGCTCCAACTACC | CGCTGCGTAAAATCTCCA | JQ793896 |
| *myf5* | GGAGAGCCGCCACTATGA | GCAGTCAACCATGCTTTCAG | GU290227 |
| *myhc* | ACGCTCATCACCACCAACCC | CAGCCTCCTCTGTGCCATCA | EU414733 |
| *mstn* | GCAGGAGTCACGTCTTGGCA | GAGTCCCTCCGGATTCGCTT | KM874826 |
| *β-actin* | GGCTGTGCTGTCCCTGTA | GGGCATAACCCTCGTAGAT | M25013 |

**Table S3** Target proteins, dilution factor, antibody Cat. No. and antibody source of proteins selected for analyzing by western blotting

| **Indices** | **Host** | **Source** | **Catalog No.** | **Dilution** |
| --- | --- | --- | --- | --- |
| MyoG | Rabbit | Affinity (Golden, Colorado, USA) | DF8273 | 1:1000 |
| Myf5 | Rabbit | Abclonal (Wuhan, Hubei, CN) | A16227 | 1:1200 |
| MyoD | Rabbit | Affinity (Golden, Colorado, USA) | AF7733 | 1:1500 |
| MYHC | Rabbit | DSHB (Iowa City, IA, USA) | MF20 | 1:50(WB)/1:5(IF) |
| T-TOR  p-TORSer488  T-S6K1  p-S6K1Ser389  T-4E-BP1  p-4E-BP1Thr37/46  β-actin  FBOX32/ MAFBX MuRF1  FoxO3  UB  FoxO1  Secondary antibody | Rabbit  Rabbit  Rabbit  Rabbit  Rabbit  Rabbit  Rabbit  Rabbit  Rabbit  Rabbit  Rabbit  Rabbit  Rabbit | Affinity (Golden, Colorado, USA)  Affinity (Golden, Colorado, USA)  Affinity (Golden, Colorado, USA)  Affinity (Golden, Colorado, USA)  Affinity (Golden, Colorado, USA)  Affinity (Golden, Colorado, USA)  Affinity (Golden, Colorado, USA)  Abclonal (Wuhan, Hubei, CN)  Abclonal (Wuhan, Hubei, CN)  Abclonal (Wuhan, Hubei, CN)  Abclonal (Wuhan, Hubei, CN)  Abclonal (Wuhan, Hubei, CN)  Affinity (Golden, Colorado, USA) | AF6308  AF3308  AF6226  AF3228  AF6432  AF3830  AF7018  A6825  A3101  AP0648  A18185  AP0127  S0001 | 1:1000  1:1000  1:1500  1:1300  1:1500  1:1200  1:3000  1:1000  1:1000  1:1000(WB)/1:100(IF)  1:1000  1:1000  1:8000 |

**Table S4** Ingredients and nutritional composition of the basal diet

| **Ingredients** | **g/kg** | **Nutrient content** | **g/kg** |
| --- | --- | --- | --- |
| Fish meal | 7.000 | Crude protein^d^ | 32.3 |
| Casein | 26.709 | Crude fat^d^ | 4.96 |
| Gelatin | 6.000 | Effective phosphorus ^f^ | 0.84 |
| Alpha-starch | 28.000 | n-3^e^ | 1.04 |
| Rice flour | 15.005 | n-6^e^ | 0.96 |
| Fish oil | 2.371 | n-3:n-6 | 1.08 |
| Soybean oil | 1.790 |  |  |
| Microcrystalline cellulose | 5.000 |  |  |
| Vitamin premix^a^ | 1.000 |  |  |
| Mineral premix^b^ | 2.000 |  |  |
| Choline chloride premix | 1.000 |  |  |
| Calcium dihydrogen phosphate | 3.000 |  |  |
| BHA | 0.015 |  |  |
| L-Threonine (98.5%) | 0.110 |  |  |
| Mitigant premix^c^ | 1.00 |  |  |
| total | 100.00 |  |  |

^a^ Per kilogram of vitamin premix (g/kg): retinyl acetate (1000,000 IU/g), 0.400; cholecalciferol (500,000 IU/g), 0.320; DL-a-tocopherol acetate (50%), 40.000; menadione (96%), 0.198; cyanocobalamin (1%), 0.940; D-biotin (2%), 0.750; thiamine nitrate (98%), 0.133; meso-inositol (97%), 22.068; folic acid (95%), 0.379; niacin (99%), 2.576; ascorbyl acetate (95%) and calcium-D-pantothenate (90%), 2.778 and 4.737; riboflavin (80%), 0.775; pyridoxine hydrochloride (98%), 0.115. All raw materials were diluted to 1 kg with corn starch

^b^ Per kilogram of mineral premix (g/kg): Na_2_SeO_3_ (44.7% Se), 0.132; CuSO_4_·5H_2_O (25.0% Cu), 0.600; ZnSO_4_·H_2_O (34.5% Zn), 7.681; MnSO_4_·H_2_O (31.8% Mn), 3.098; MgSO_4_·H_2_O (15.0% Mg), 237.840; CaI_2_ (3.2% I), 1.560; FeSO_4_·H_2_O (30.0% Fe), 15.000. All raw materials were diluted to 1 kg with corn starch

^c^ OTA and Cur premix: Addition of premix to obtain graded levels of ochratoxin A and curcumin

^d^ crude protein and crude fat content: measured values

^e^ The contents of n-3 and n-6 were calculated with reference to Zeng et al. [2]

^f^ The content of available phosphorus was calculated with reference to Liang et al. [3]

**Table S5** Raw data of mitigation screening results

| Cat. No. | CAS No. | Product Name | 2.5 μmol/L | | 5 μmol/L | | 10 μmol/L | |
| --- | --- | --- | --- | --- | --- | --- | --- | --- |
|  |  |  | Cell viability, % | standard deviation | Cell viability, % | standard deviation | Cell viability, % | Standard deviation |
|  |  | DMSO (0.08%) | 100 | 5.838473818 | 100 | 7.422408097 | 100 | 5.338295215 |
|  |  | Ochratoxin A | 50.10105814 | 6.324220907 | 49.10536779 | 4.868612049 | 53.9019124 | 10.43763737 |
| HY-N0693 | 61281-38-7 | Schisandrin A | 48.3355 | 5.421462787 | 56.5976 | 2.987909047 | 48.7662 | 6.809735907 |
| HY-N0894 | 36062-07-4 | Octahydrocurcumin | 51.9320 | 6.366452848 | 55.3082 | 2.701457318 | 59.5928 | 10.453183 |
| HY-N0408 | 39012-20-9 | Picroside II | 49.8752 | 6.913037892 | 53.3996 | 7.416278665 | 56.6883 | 9.886861163 |
| HY-N0649 | 604-80-8 | Narcissin | 51.7180 | 7.088355867 | 57.3985 | 4.61030907 | 60.0966 | 13.41910345 |
| HY-N0640 | 7084-24-4 | Kuromanin (chloride) | 73.2493 | 10.46470284 | 82.9821 | 8.936214616 | 71.9669 | 9.74323236 |
| HY-N0148 | 153-18-4 | Rutin | 56.6758 | 14.49445359 | 85.9983 | 1.518242906 | 78.9225 | 13.55667915 |
| HY-N0342 | 92-61-5 | Scopoletin | 61.1937 | 17.06498951 | 78.6652 | 11.04666635 | 69.3810 | 4.624293101 |
| HY-N0197 | 21967-41-9 | Baicalin | 45.7318 | 8.851765713 | 46.4414 | 1.939866584 | 54.9455 | 7.142990419 |
| HY-N0451 | 480-44-4 | Acacetin | 45.6486 | 8.852941212 | 51.0026 | 9.76763868 | 52.5704 | 4.859350696 |
| HY-N2065 | 5119-48-2 | Withaferin A | 53.4776 | 5.321354133 | 49.4405 | 10.9014946 | 57.4234 | 16.6379789 |
| HY-N0368 | 78-70-6 | Linalool | 62.1032 | 4.986983501 | 47.9580 | 12.63904294 | 59.0531 | 12.61277103 |
| HY-Y0248A | 4602-84-0 | Farnesol | 52.8118 | 9.612859397 | 51.8319 | 11.34426462 | 57.5005 | 7.241567361 |
| HY-N0722 | 906-33-2 | Neochlorogenic acid | 48.3712 | 4.613249865 | 51.1218 | 15.38401852 | 59.7008 | 12.78536587 |
| HY-N0328 | 6147-11-1 | alpha-Mangostin | 56.5569 | 7.30751131 | 50.8833 | 12.40278233 | 51.6862 | 10.69635029 |
| HY-N0036 | 553-21-9 | Costunolide | 47.5746 | 4.645177778 | 45.1519 | 12.33761775 | 54.9455 | 6.640909028 |
| HY-N0893 | 36062-04-1 | Tetrahydrocurcumin | 46.9677 | 8.256271199 | 48.8952 | 8.275795628 | 54.7964 | 13.40874371 |
| HY-B0075 | 73-31-4 | Melatonin | 46.3144 | 4.698869189 | 53.3882 | 8.131996049 | 57.0276 | 11.66848125 |
| HY-N0767 | 4261-42-1 | Isoorientin | 71.5670 | 11.64237812 | 87.4922 | 10.57564252 | 71.0415 | 10.97811833 |
| HY-N0683 | 59-02-9 | α-Vitamin E | 67.3642 | 9.856654925 | 79.2105 | 11.553844 | 69.7255 | 11.68477696 |
| HY-W001179 | 490-79-9 | 2,5-Dihydroxybenzoic acid | 51.2008 | 7.585782295 | 50.7356 | 3.181639579 | 52.5499 | 8.488850205 |
| HY-B1030 | 17575-22-3 | Lanatoside C | 47.5568 | 7.525056996 | 48.9066 | 12.37588323 | 52.2003 | 8.962083218 |
| HY-N0790 | 545-47-1 | Lupeol | 52.2946 | 4.666440361 | 53.0020 | 5.336223122 | 54.3749 | 11.63258178 |
| HY-N0516 | 479-91-4 | Casticin | 43.7582 | 4.081124411 | 51.6728 | 14.09678492 | 54.4314 | 6.294301765 |
| HY-N6810 | 89-83-8 | Thymol | 48.0383 | 7.532227818 | 46.7026 | 12.34920665 | 54.1847 | 9.658243563 |
| HY-N4288 | 529-84-0 | 4-Methylesculetin | 45.6961 | 6.106199161 | 49.2928 | 10.39423938 | 49.2032 | 7.457545725 |
| HY-N0235 | 10309-37-2 | Bakuchiol | 48.5139 | 4.588310275 | 51.7694 | 11.1956268 | 51.7839 | 5.895281533 |
| HY-N0619 | 102841-42-9 | Mulberroside A | 43.3480 | 3.949755819 | 54.5300 | 1.723828685 | 55.6395 | 5.362277425 |
| HY-30151 | 298-81-7 | Methoxsalen | 44.8936 | 4.745646797 | 52.6214 | 1.909201955 | 52.7915 | 8.987012876 |
| HY-N0580 | 574-84-5 | Fraxetin | 51.3970 | 10.06494554 | 50.7867 | 7.666985746 | 52.6116 | 10.71117088 |
| HY-18085 | 117-39-5 | Quercetin | 45.6010 | 4.724160797 | 47.6342 | 5.438553308 | 53.0023 | 11.94658339 |
| HY-N1987 | 50298-90-3 | Cucurbitacin IIb | 48.2345 | 6.888804651 | 44.9361 | 6.799582208 | 50.3650 | 9.742972542 |
| HY-N0523 | 149-91-7 | Gallic acid | 50.4518 | 8.3246313 | 50.6021 | 4.732341819 | 47.2445 | 10.59202551 |
| HY-N1390 | 134-96-3 | Syringaldehyde | 52.5562 | 7.375525009 | 48.3329 | 2.943803865 | 50.0617 | 8.692824482 |
| HY-N0112 | 27200-12-0 | Dihydromyricetin | 42.6763 | 3.940915429 | 51.8091 | 3.745308014 | 47.9437 | 5.528066945 |
| HY-N0105 | 478-43-3 | Rhein | 48.0799 | 6.929243762 | 50.0710 | 6.283300334 | 45.9901 | 4.152785316 |
| HY-B0094 | 63968-64-9 | Artemisinin | 50.6063 | 7.710713446 | 49.5995 | 7.834045123 | 50.0463 | 9.82016551 |
| HY-N0337 | 97-53-0 | Eugenol | 51.9558 | 7.10595042 | 83.4138 | 15.75260346 | 69.7923 | 6.198162095 |
| HY-N6804 | 79165-06-3 | Diammonium Glycyrrhizinate | 48.6981 | 9.652971964 | 93.6723 | 13.57875131 | 76.3161 | 13.02603937 |
| HY-N0088 | 498-02-2 | Apocynin | 50.5291 | 8.316737512 | 61.0452 | 16.50719729 | 45.8462 | 3.935797676 |
| HY-N0376 | 551-15-5 | Liquiritin | 48.1393 | 9.654364682 | 58.2221 | 9.52421089 | 43.1472 | 2.75798482 |
| HY-N0509 | 29838-67-3 | Astilbin | 47.0277 | 4.67367716 | 64.6237 | 13.75327762 | 47.3525 | 3.636520139 |
| HY-N0001 | 490-46-0 | (-)-Epicatechin | 50.4340 | 7.509352125 | 61.4939 | 14.219237 | 48.8484 | 5.418115112 |
| HY-N0494 | 20831-76-9 | Gentiopicroside | 52.0390 | 7.098170599 | 73.3826 | 6.119392572 | 51.7582 | 8.881035287 |
| HY-N0106 | 67920-52-9 | Danshensu (sodium salt) | 46.3500 | 5.321773771 | 52.2352 | 5.69325846 | 49.0181 | 4.975177993 |
| HY-N0013 | 3681-93-4 | Vitexin | 47.2833 | 10.98813943 | 53.1497 | 6.958021225 | 53.6397 | 10.43315131 |
| HY-N0290 | 4773-96-0 | Mangiferin | 48.1393 | 7.815360218 | 47.6683 | 6.179503227 | 52.1643 | 8.950461492 |
| HY-N0578 | 578-74-5 | Apigenin 7-glucoside | 47.5865 | 6.936897754 | 55.0809 | 9.595889186 | 53.1719 | 5.597561899 |
| HY-N0441 | 2292-16-2 | Neferine | 49.2272 | 8.587916295 | 52.6498 | 2.630754475 | 46.9155 | 6.334469551 |
| HY-N0529 | 20283-92-5 | Rosmarinic acid | 49.5720 | 9.335268242 | 53.0133 | 2.642538281 | 44.7769 | 4.135852068 |
| HY-N0563 | 72-48-0 | Alizarin | 48.5792 | 7.402317289 | 48.7589 | 2.533527813 | 54.2669 | 4.557512356 |
| HY-N0705 | 85643-19-2 | Curculigoside | 47.7173 | 7.392897007 | 52.5589 | 2.630754475 | 54.9249 | 11.7470838 |
| HY-N0633 | 541-91-3 | Muscone | 50.3745 | 4.721679664 | 52.6725 | 2.630754475 | 53.1771 | 14.4016355 |
| HY-N1425 | 20316-62-5 | Tiliroside | 44.0970 | 4.778096968 | 57.1713 | 2.748592554 | 50.9973 | 4.940418533 |
| HY-N0139 | 7085-55-4 | Troxerutin | 42.1947 | 4.84728693 | 53.3882 | 7.094312784 | 52.4728 | 5.959201687 |
| HY-N1454 | 29741-09-1 | Apigenin-7-glucuronide | 48.2761 | 3.968740932 | 57.3530 | 2.760376363 | 48.2367 | 6.035669394 |
| HY-15097 | 529-44-2 | Myricetin | 42.7179 | 5.66891677 | 54.1721 | 12.82027099 | 46.3140 | 5.98736029 |
| HY-N0005 | 458-37-7 | Curcumin | 84.2409 | 4.899426278 | 127.7364 | 4.821128731 | 106.2873 | 6.951297827 |
| HY-N0779 | 72581-71-6 | Isosilybin | 52.0866 | 5.640809321 | 86.0721 | 1.219126735 | 78.6089 | 9.793420381 |
| HY-N0344 | 24211-30-1 | Farrerol | 53.2220 | 10.07316061 | 53.8256 | 4.683617267 | 54.4777 | 12.57217475 |
| HY-N0645 | 66-76-2 | Dicoumarol | 54.5655 | 6.57361755 | 51.2582 | 2.592447065 | 53.0845 | 3.433674417 |
| HY-N0359 | 30964-13-7 | Cynarin | 47.6697 | 9.660574618 | 57.5802 | 2.760376363 | 51.5885 | 8.505068306 |
| HY-N0822 | 517-89-5 | Shikonin | 52.5027 | 10.06166953 | 54.4277 | 2.680825942 | 57.9735 | 9.25836425 |
| HY-N0021 | 61276-17-3 | Verbascoside | 54.7319 | 7.176991172 | 50.6390 | 11.02645693 | 57.0944 | 9.053821071 |
| HY-N0598 | 53963-43-2 | Ginsenoside F1 | 54.9637 | 7.244279047 | 45.0156 | 11.03031294 | 59.9013 | 9.310223969 |
| HY-N0472 | 60976-49-0 | Geraniin | 44.9649 | 4.745646797 | 50.3664 | 11.02354435 | 58.0095 | 11.89999917 |
| HY-N0859 | 69363-14-0 | Schisanhenol | 56.4618 | 7.30751131 | 51.9398 | 11.02419975 | 58.6007 | 6.656992047 |
| HY-N0136 | 480-18-2 | Taxifolin | 46.7364 | 4.558060221 | 55.3309 | 2.701457318 | 56.4158 | 11.88261985 |
| HY-N6972 | 481-49-2 | Cepharanthine | 44.8758 | 3.375724161 | 53.7518 | 2.660213991 | 57.5365 | 15.07545163 |
| HY-32736 | 38647-11-9 | Triptonide | 44.6736 | 6.172020084 | 57.5234 | 9.535446496 | 58.5647 | 6.611556308 |
| HY-N0109 | 10338-51-9 | Salidroside | 50.2497 | 7.276017659 | 63.9761 | 7.837243294 | 59.0788 | 14.93816058 |
| HY-N0215 | 63-91-2 | L-Phenylalanine | 44.1386 | 2.437106406 | 63.8455 | 6.065822069 | 58.6058 | 8.6901142 |
| HY-W018643 | 2309-07-1 | Ferulic acid methyl ester | 42.2364 | 2.020277569 | 44.8111 | 9.560606281 | 50.2673 | 7.131414041 |
| HY-N0271 | 510-30-5 | Echinocystic acid | 43.2232 | 4.814561938 | 51.5990 | 9.296180837 | 58.9708 | 8.997428856 |
| HY-N0318 | 96574-01-5 | Salvianolic acid A | 53.2993 | 7.161407049 | 106.5834 | 5.798624275 | 78.3673 | 7.211161432 |
| HY-B0183 | 476-66-4 | Ellagic acid | 52.6691 | 7.138584683 | 82.6470 | 12.37588323 | 91.9134 | 6.363130695 |
| HY-N0629 | 4373-41-5 | Maslinic acid | 52.9901 | 7.148407745 | 78.3243 | 15.37060172 | 71.1187 | 7.326847957 |
| HY-N0828 | 537-42-8 | Pterostilbene | 54.3336 | 7.201824301 | 70.6390 | 12.36251704 | 83.0516 | 7.123378156 |
| HY-17577 | 68030-18-2 | Berberine (chloride hydrate) | 53.3825 | 7.171224531 | 50.2357 | 7.834466082 | 54.4160 | 12.10246179 |
| HY-N0194 | 464-92-6 | Asiatic acid | 53.8997 | 4.184183296 | 56.2454 | 7.394366059 | 59.5157 | 11.44672866 |
| HY-N6029 | 111664-82-5 | Dehydroevodiamine (hydrochloride) | 52.5918 | 7.132406121 | 46.3845 | 9.52421089 | 54.9455 | 6.043763843 |
| HY-13067 | 34157-83-0 | Tripterin | 53.9413 | 5.299017955 | 45.1519 | 9.54561656 | 45.6920 | 4.946108491 |
| HY-N0570 | 10597-60-1 | Hydroxytyrosol | 44.9649 | 4.745646797 | 55.2684 | 6.082182133 | 53.1102 | 5.872934295 |
| HY-N7510 | 65666-07-1 | Carnosic acid | 49.0013 | 4.59518917 | 49.2758 | 9.320261593 | 48.9101 | 5.363670538 |
| HY-N0794 | 20347-71-1 | Proanthocyanidins | 44.5666 | 4.756505866 | 44.4306 | 11.02943365 | 43.8978 | 3.888810714 |
| HY-N0029 | 81525-13-5 | Forsythoside B | 41.4754 | 7.707010272 | 44.4306 | 11.02943365 | 53.1308 | 8.112277343 |
| HY-N0089 | 61281-37-6 | Schisandrin B | 43.4193 | 3.949755819 | 48.5430 | 10.58312607 | 48.0465 | 5.721610997 |
| HY-N0168 | 520-33-2 | Hesperetin | 48.9716 | 3.90218687 | 46.6118 | 11.02754501 | 49.5784 | 6.501844555 |
| HY-N1976 | 225110-25-8 | (+)-(3R,8S)-Falcarindiol | 48.2701 | 7.404431858 | 46.4527 | 11.02754501 | 50.2313 | 6.775737836 |
| HY-16561 | 501-36-0 | Resveratrol | 52.9782 | 10.07273779 | 45.5552 | 13.51757102 | 50.9819 | 12.07202468 |
| HY-B1461 | 53936-56-4 | Deoxyarbutin | 55.9862 | 10.09166284 | 54.4050 | 2.680825942 | 54.2875 | 10.87010231 |
| HY-N0201 | 73069-13-3 | Atractylenolide I | 47.0099 | 10.20039636 | 48.8838 | 9.485862251 | 54.5394 | 11.0763324 |
| HY-N0110 | 10605-02-4 | Palmatine (chloride) | 45.3632 | 5.608609623 | 48.4067 | 8.961800211 | 47.5067 | 3.174187319 |
| HY-N0196 | 491-67-8 | Baicalein | 56.3845 | 10.0905883 | 45.2599 | 9.543463831 | 50.1080 | 4.32425626 |
| HY-N7073 | 22888-70-6 | Silymarin | 46.5878 | 5.425928216 | 50.5425 | 9.270425127 | 50.3907 | 9.380028761 |
| HY-B2163 | 61281-38-7 | Schisandrin A | 51.3970 | 6.960325233 | 52.3931 | 1.850760044 | 56.3181 | 7.342491529 |


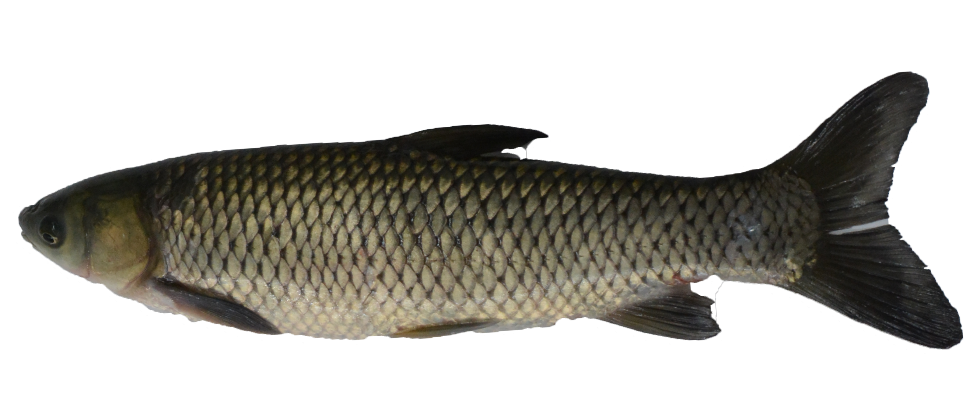


M1

M2

**Fig. S1** Schematic diagram of muscle sampling sites for grass carp


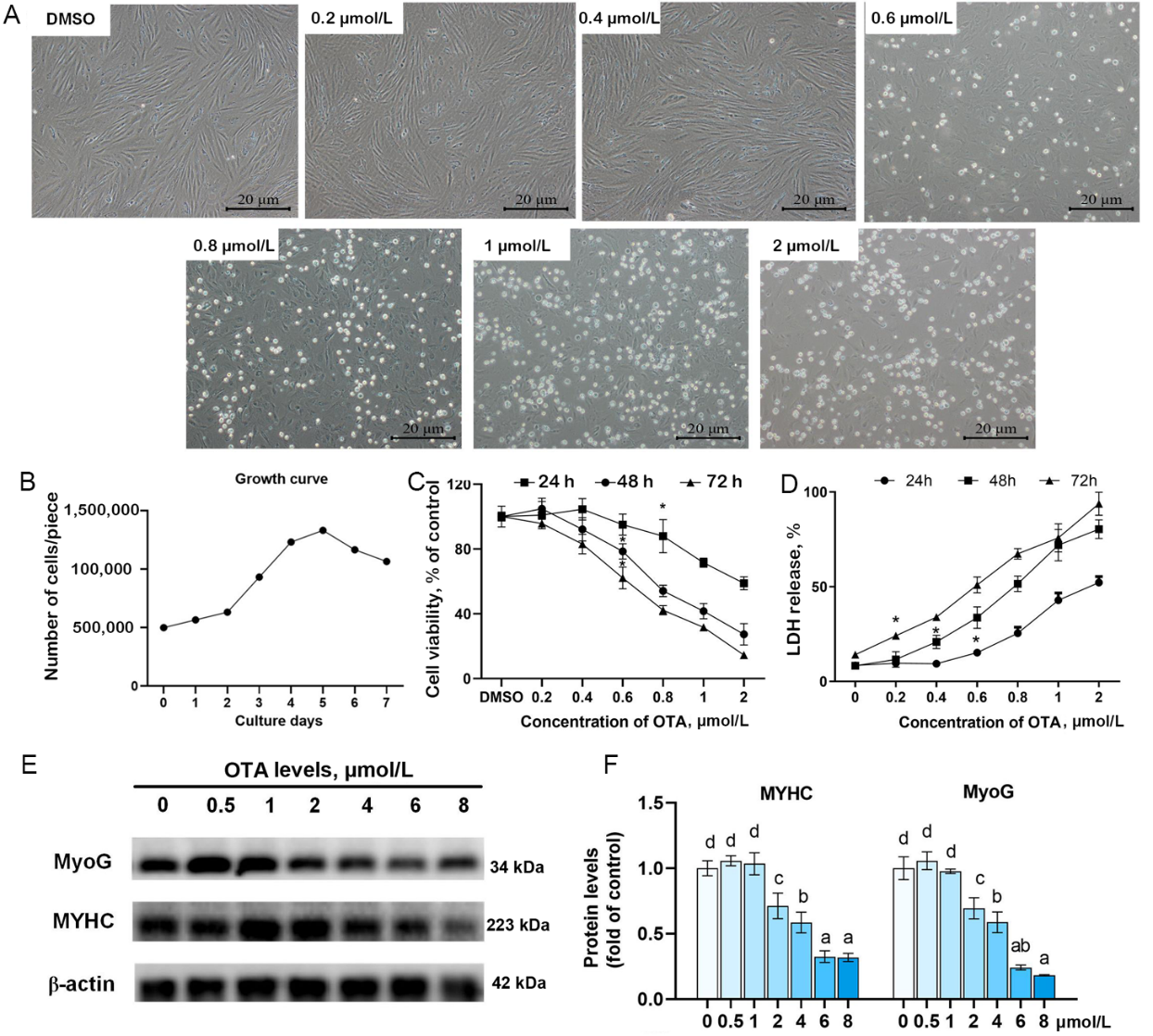


**Fig. S2** Determination of the dose of OTA inhibiting the proliferation and differentiation of primary myoblasts in grass carp. **A–B** The 7d growth curve of primary myoblasts of grass carp and cell morphology changes at 48 h after exposure to different doses of OTA, *n* = 6, ×200; **C–D** The effects of different doses of OTA on cell viability and LDH at 24, 48, and 72 h, *n* = 6. **E–F** Dose determination of OTA inhibited the protein expression of MYHC and MyoG, *n* = 3. For all quantifications, data (mean ± SD) were from the indicated number of independent experiments. Six treatments were performed by one-way ANOVA, different lowercase letters between different processes indicate significant differences (*P* < 0.05) [4]


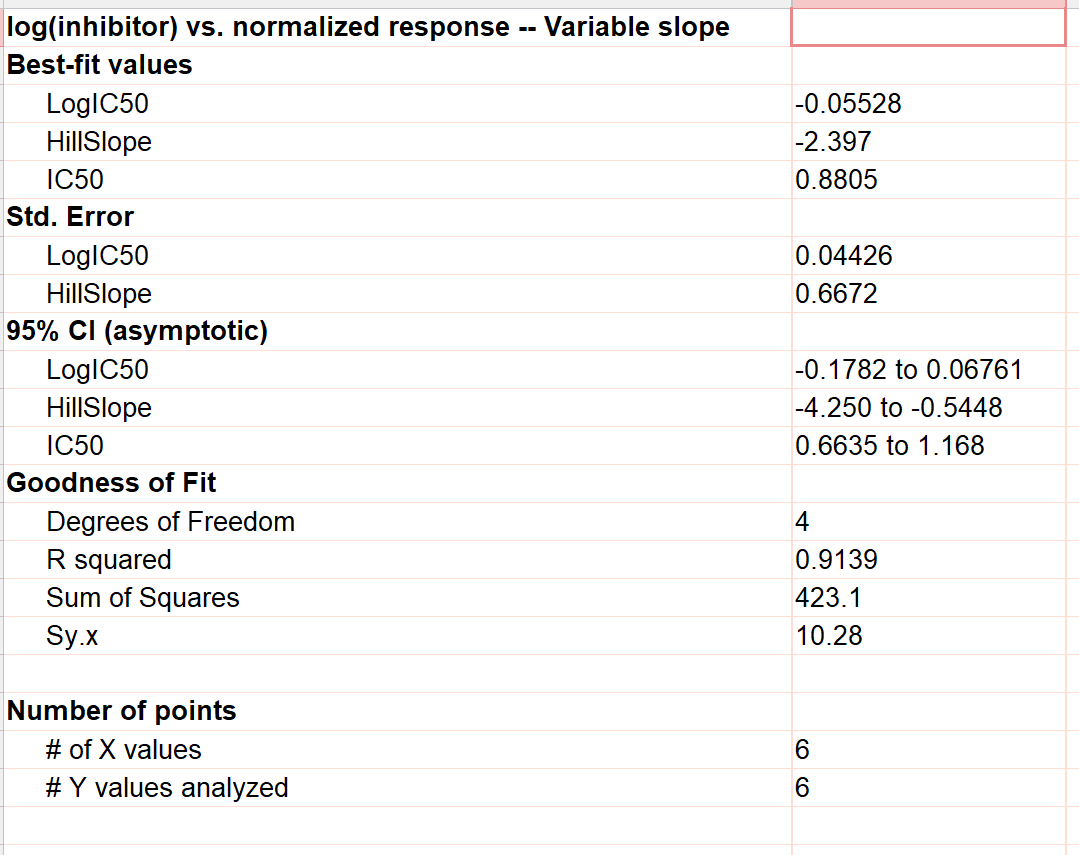


**Fig. S3** IC50 results of OTA cytotoxicity to grass carp primary myoblast were calculated by Graphpad 8 [4]


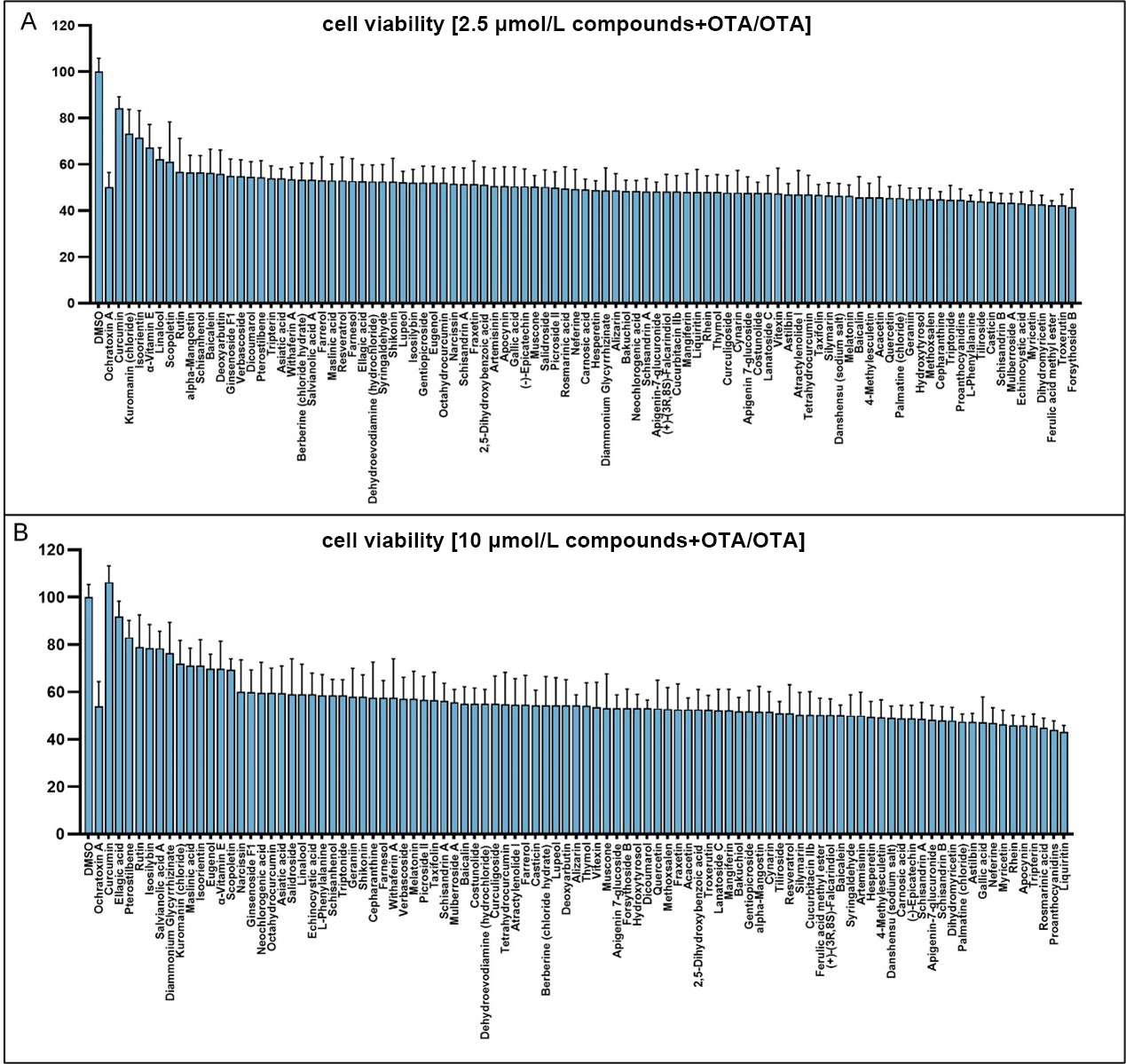


**Fig. S4** Anti-OTA inhibits cell viability of natural product. Grass carp primary myoblasts were treated with OTA (0.8 μmol/L) in the absence or presence of the indicated natural product compounds (2.5 and 10 μmol/L). Cell viability was assayed using the CCK-8 kit, *n* = 3

**Reference**

1. Zhou Y, Wu P, Feng L, Jiang WD, Liu Y, Peng Y, et al. Dietary cinnamaldehyde improves muscle protein content by promoting muscle fiber growth via PTP1B/IGF1/PI3K/AKTs-TOR/FOXO3a signaling pathway in grass carp (*Ctenopharyngodon idella*). Food Chem, 2023;399:133799. https://doi.org/10.1016/j.foodchem.2022.133799.

2. Zeng YY, Feng L, Jiang WD, Liu Y, Wu P, Jiang J, et al. Dietary alpha-linolenic acid/linoleic acid ratios modulate immune response, physical barrier and related signaling molecules mRNA expression in the gills of juvenile grass carp (*Ctenopharyngodon idella*). Fish Shellfish Immunol. 2016;62,1-12. https://doi.org/10.1016/j.fsi.2017.01.003.

3. Liang JJ, Liu Y J, Tian LX, Yang HJ, Liang JY. Dietary available phosphorus requirement of juvenile grass carp (*Ctenopharyngodon idella*). Aquac Nutr. 2012;18(2):181-188. https://doi.org/10.1111/j.1365-2095.2011.00887.x.

4. Zhao P, Zhang L, Feng L, Jiang WD, Wu P, Liu Y, et al. Novel perspective on mechanism in muscle growth inhibited by ochratoxin A associated with ferroptosis: Model of juvenile grass carp (*Ctenopharyngodon idella*) in vivo and in vitro trials. J Agr Food Chem. 2024. https://doi.org/10.1021/acs.jafc.3c08080.
